# Glucosylceramide in bunyavirus particles is essential for virus binding to host cells

**DOI:** 10.1007/s00018-023-05103-0

**Published:** 2024-02-01

**Authors:** Zina M. Uckeley, Maëva Duboeuf, Yu Gu, Alexandra Erny, Magalie Mazelier, Christian Lüchtenborg, Sophie L. Winter, Paulina Schad, Cyrille Mathieu, Jana Koch, Steeve Boulant, Petr Chlanda, Carine Maisse, Britta Brügger, Pierre-Yves Lozach

**Affiliations:** 1 Center for Integrative Infectious Diseases Research (CIID), University Hospital Heidelberg, 69120 Heidelberg, Germany; 2 Cluster of Excellence, CellNetworks, 69120 Heidelberg, Germany; 3 Department of Infectious Diseases, Virology, University Hospital Heidelberg, 69120 Heidelberg, Germany; 4 Department for Molecular Genetics and Microbiology, University of Florida, Gainesville, USA; 5 Université Claude Bernard Lyon 1, INRAE, EPHE, IVPC UMR754, Team iWays, 69007 Lyon, France; 6 Heidelberg University Biochemistry Center (BZH), Heidelberg, Germany; 7 Schaller Research Groups, Department of Infectious Diseases, Virology, Heidelberg University Hospital, Heidelberg, Germany; 8 CIRI (Centre International de Recherche en Infectiologie), Team Neuro-Invasion, TROpism and VIRal Encephalitis, INSERM U1111, CNRS UMR5308, Université Claude Bernard Lyon 1, Ecole Normale Supérieure de Lyon, 69007 Lyon, France

**Keywords:** Alphavirus, Glucosylceramide synthase, Lipidomics, UGCG, Virus–receptor interactions

## Abstract

Hexosylceramides (HexCer) are implicated in the infection process of various pathogens. However, the molecular and cellular functions of HexCer in infectious cycles are poorly understood. Investigating the enveloped virus Uukuniemi (UUKV), a bunyavirus of the *Phenuiviridae* family, we performed a lipidomic analysis with mass spectrometry and determined the lipidome of both infected cells and derived virions. We found that UUKV alters the processing of HexCer to glycosphingolipids (GSL) in infected cells. The infection resulted in the overexpression of glucosylceramide (GlcCer) synthase (UGCG) and the specific accumulation of GlcCer and its subsequent incorporation into viral progeny. UUKV and several pathogenic bunyaviruses relied on GlcCer in the viral envelope for binding to various host cell types. Overall, our results indicate that GlcCer is a structural determinant of virions crucial for bunyavirus infectivity. This study also highlights the importance of glycolipids on virions in facilitating interactions with host cell receptors and infectious entry of enveloped viruses.

**Supplementary Information:**

The online version contains supplementary material available at 10.1007/s00018-023-05103-0.

## Introduction

The *Bunyavirales* is a large order of RNA viruses, which comprises a dozen viral families [1]. With more than 500 members, these viruses represent a global threat to human public health and agricultural productivity. Many are causes of birth defects and serious diseases in both humans and livestock, such as acute hepatitis, hemorrhagic fever, and encephalitis. Bunyaviruses infect a large spectrum of hosts, including vertebrates, invertebrates, and plants [2]. Except for arenaviruses and hantaviruses, all the other bunyaviruses are transmitted by arthropods and therefore belong to the supergroup of arthropod-borne viruses (arboviruses). Owing to their mode of transmission, global warming, and the increasing number of recent outbreaks worldwide, bunyaviruses are considered potential agents of emerging diseases. To date, no vaccines or specific antiviral treatments have been approved for human use.

At the molecular level, bunyaviral particles are enveloped and roughly spherical (approximately 80–120 nm in diameter) with mainly tripartite single-stranded RNA genome that replicates exclusively in the cytosol of infected cells [3, 4]. The viral structural proteins are encoded in the negative sense orientation with the nucleoprotein N encoded in the small genomic segment (S), the transmembrane envelope glycoproteins encoded in the medium segment (M), and the RNA-dependent RNA polymerase (RdRp) encoded in the large segment (L). The viral particles assemble and bud in the Golgi, where they acquire a lipid bilayer envelope and exit from infected cells [5]. Within virions, the N protein is associated with the RNA genome and, together with the viral RdRp, forms ribonucleoproteins (RNPs) [6]. The viral glycoproteins protrude from the viral surface and ensure the penetration of virions into host cells [7, 8].

The transmission, tropism, and cell entry remain poorly characterized for a large majority of bunyaviruses although many attachment factors have been characterized [2–4, 9]. In most cases, whether the factors serve as a true endocytic receptor, attachment factor, or cofactor is not known. Nevertheless, there is increasing evidence that bunyaviruses rely on a variety of receptors to target and bind different host cell tissues. An illustration is the human C-type lectins dendritic cell-specific intercellular adhesion molecule 3-grabbing nonintegrin (DC-SIGN) and liver/lymph node cell-specific intercellular adhesion molecule 3-grabbing nonintegrin (L-SIGN). Both lectins capture various bunyaviruses via mannose residues carried by the viral envelope glycoproteins and are interesting candidates to explain the tropism of bunyaviruses for dermal dendritic cells and liver [10–13]. After attachment, bunyaviruses are sorted into the endocytic machinery and penetrate host cells via acid-activated membrane fusion [3, 4, 14].

All the virus—receptor interactions described so far as responsible for the attachment of bunyaviruses to target cells occur through the recognition of viral glycoproteins decorating virions by cell surface receptors. Interestingly, viral envelope lipids have been reported to modulate binding and thus regulate the tropism of unrelated viruses. Transmembrane immunoglobulin and mucin domain (TIM) and Tyro3, Axl, and Mer (TAM) proteins, two families of receptors mediating phosphatidylserine (PS)-dependent phagocytosis, are receptors of flaviviruses and filoviruses [15]. Although a specific interaction between PS in viral envelopes and TIM/TAM remains to be identified, previous observations suggest that viral envelope lipids are important binding modulators. Overall, the lipid composition of enveloped viruses and their functional significance remain to be sufficiently characterized, and little information is available on the importance of lipids to bunyavirus entry.

We focus here on Uukuniemi virus (UUKV), a bunyavirus of the family *Phenuiviridae*. UUKV is thus related to Toscana virus (TOSV), Rift Valley fever virus (RVFV), Heartland virus (HRTV), Dabie virus (DABV), previously referred to as severe fever with thrombocytopenia syndrome virus (SFTSV), and other highly pathogenic phenuiviruses that have recently emerged in China, North America, and Europe [16]. To examine the roles played by lipids in early bunyavirus—host cell interactions, we subjected UUKV particles produced in mammalian tissue culture cells to a lipidomic analysis with mass spectrometry (MS). Using this approach, we identified the glycolipid glucosylceramide (GlcCer), which is a transient intermediate in glycosphingolipid (GSL) synthesis, as a crucial viral factor for the attachment of UUKV and other bunyaviruses to various cell types. Overall, our results clarify the role of GlcCer in the infectious cycle of several bunyaviruses and shed new light on how these viruses manipulate the GSL pathway.

## Results

### A quantitative lipidomic analysis led to the identification of hexosylceramide (HexCer) as a significant component of the UUKV particles

The lipids that constitute bunyaviruses have not been extensively documented. To better understand the role played by lipids in the viral envelope on bunyavirus—host cell interactions, we first employed a lipidomic analysis with MS approach to determine the lipid distribution in cells after UUKV infection. To this end, baby hamster kidney fibroblasts (BHK-21 cells) were exposed to UUKV and harvested 48 h post-infection, after which several cycles of infection occurred and virus progeny production is peaking [7]. At this time, cells were still alive as determined with a quantitative assay measuring the release of lactate dehydrogenase (LDH) into the extracellular medium upon cell death and lysis (Figure S1). BHK-21 cells constitute the gold standard model system to study UUKV and other bunyaviruses. In these cells, the detection of newly-synthesized UUKV proteins is possible 1 h post-infection, and a complete UUKV life cycle, from infection to release of infectious progeny, lasts 5–7 h [7].

**Fig. 1:**
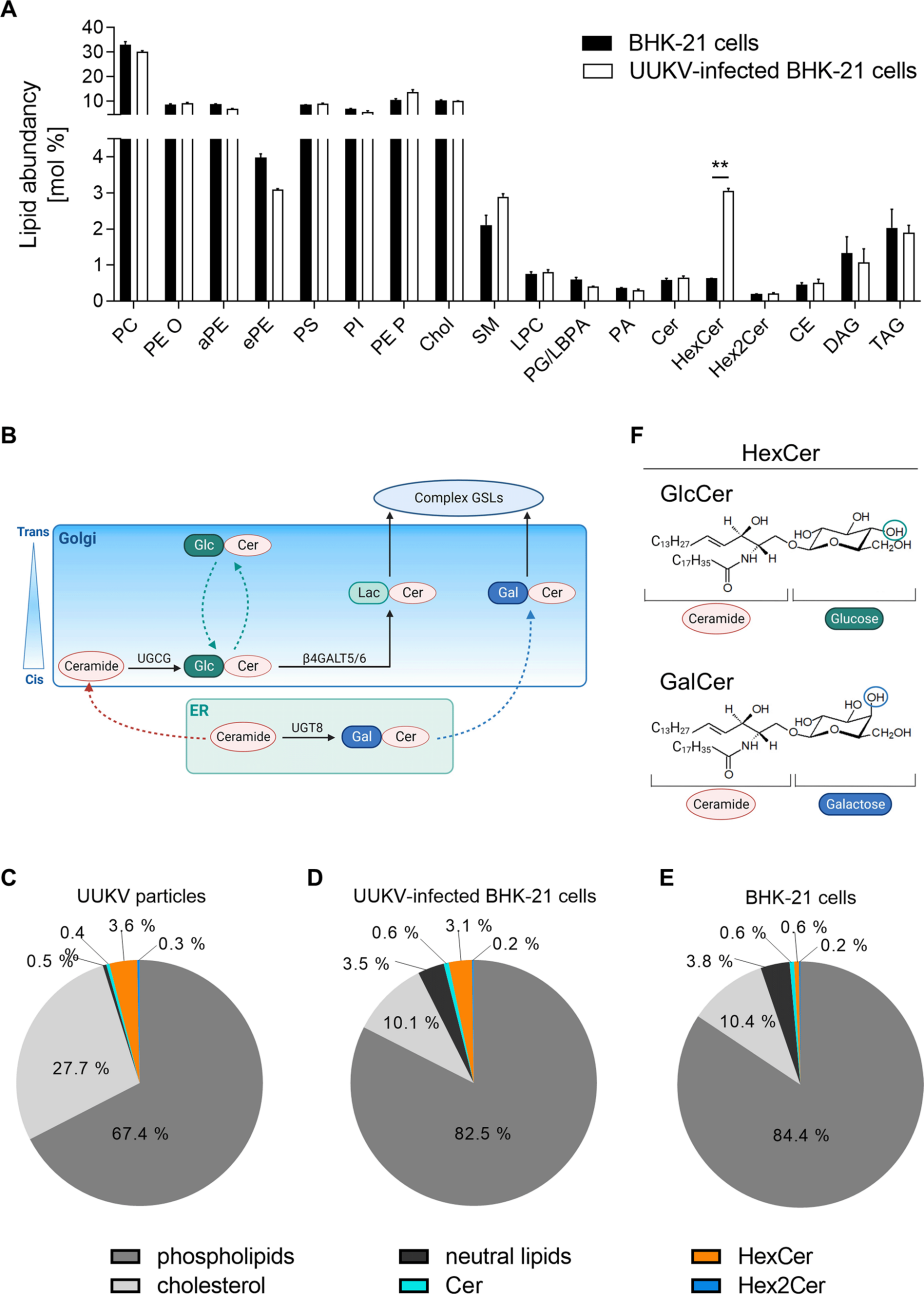
A label-free quantitative lipidomic analysis led to the identification of hexosylceramide (HexCer) as a major component of Uukuniemi virus (UUKV) particles. **A** BHK-21 cells were infected with UUKV at a multiplicity of infection of 0.1 for 48 h and then subjected to a lipidomic analysis. Lipid classes and subclasses are presented as mol % fractions. Unpaired t test with Welch correction was applied (*n* = 3). **, *p* < 0.01. **B** Schematic overview showing HexCer metabolism. The dashed colored lines indicate intracellular trafficking, while the dark lines show enzymatic reactions. β4GALT5/6, LacCer synthases β4GALT5 and β4GALT6; ER, endoplasmic reticulum; GalCer, galactosylceramide; GlcCer, glucosylceramide; GSL, glycosphingolipid; UGCG, UDP − GlcCer glucosyltransferase; UGT8, UDP − GalCer galactosyltransferase. Created with BioRender.com. **C** Supernatant from infected BHK-21 cells was harvested 48 h post-infection, and UUKV particles were purified before lipid MS analysis (*n* = 3). **D** Lipidome of UUKV-infected BHK-21 cells (*n* = 3). **E** Lipidome of uninfected BHK-21 cells (*n* = 3). **C–E** The phospholipids included all glycerophospholipids such as phosphatidylethanolamine plasmalogen (PE P), SM, and lysophosphatidylcholine (LPC). The neutral lipids were cholesteryl ester (CE), diacylglycerol (DAG), and triacylglycerol (TAG). **F** Chemical formulas of GlcCer and GalCer. Created with BioRender.com

Infected cells were subjected to a quantitative MS-based lipid analysis applying a shotgun lipidomic approach [17]. We quantitatively assessed 374 lipid species in 22 lipid classes and subclasses, including the glycerophospholipids phosphatidylcholine (PC), lyso-PC (LPC), phosphatidylethanolamine (PE), phosphatidylinositol (PI), PS, phosphatidic acid (PA), PE plasmalogen (PE P), and phosphatidylglycerol/lysobisphosphatidic acid (PG/LBPA) (Table S1). PC, PE, PS, PI, and PG were further subcategorized into diacyl designated without a prefix, indicating the type and species with either plasmanyl/acyl or diacyls, and with the prefix O, indicating even/odd chain fatty acyl composition. A sphingolipid species analysis was performed to detect sphingomyelin (SM), ceramide (Cer), HexCer, and dihexosylceramide (Hex2Cer) species. The sterol lipids identified included cholesterol (Chol) and cholesteryl ester (CE) species, and triacylglycerol (TAG) and diacylglycerol (DAG) were representative neutral lipids. The results showed that infection did not affect the distribution of the lipid classes in producer cells, except HexCer, the level of which was increased fivefold compared with that of uninfected cells (Fig. 1A, Table S1). HexCers comprise GlcCer and galactosylceramide (GalCer) that are glycolipids in the endoplasmic reticulum (ER) and Golgi apparatus, where they are transient intermediates in GSL synthesis (Fig. 1B) [18]. The MS analysis revealed that the abundance of Cer and Hex2Cer, upstream and downstream intermediates of HexCer in this GSL-producing pathway, was not affected by viral infection, as indicated by the levels of both lipids remaining at a minimal level (Fig. 1A). Taken together, our results showed that UUKV infection leads to HexCer accumulation in cells, presumably in the Golgi apparatus.

The Golgi network is the assembly site of UUKV and other bunyaviruses [5]. We, therefore, wondered whether an increase in HexCer levels leads to its incorporation into the envelope of UUKV particles. To assess this possibility, we performed a lipidomic analysis with MS of purified viral particles produced in BHK-21 cells (Figs. 1C and S2A, and Table S2). Typical Golgi resident proteins such as calnexin and TGN46 were not detected in our purified virus stocks, as assayed by western blotting (Figure S2B). This suggested that no Golgi-associated fragments were present in our samples. In general, the protein purity of our virus stocks was greater than 90%, as assayed by Coomassie blue staining (Figure S2C). In these experiments, we additionally used virus-free mock cell supernatants to control for lipid contaminants of the UUKV preparations. No significant amount of lipid was present in the virus-free mock cell supernatant after the purification process. We found that Chol and the phospholipids SM and PE P were enriched in UUKV particles compared with the total lipid composition in the producer cells (Fig. 1A, C, D, E, and S2A and Tables S1 and S2). These lipid classes are typical of the post-*cis* stacks of the Golgi network [19, 20], consistent with the budding of viral particles from this compartment. HexCers accounted for almost 4% of the lipids in the viral envelope (Fig. 1C).

Overall, our data indicated that UUKV infection modulates the GSL synthesis pathway such that HexCer is enriched in infected cells and is likely incorporated into viral particles during budding. In all further experiments, we sought to determine whether the incorporation of HexCer into the viral envelope exhibited a specific function in bunyavirus—host cell interactions.

### GlcCer is incorporated into UUKV particles

Our lipidomic analysis with MS approach did not allow us to discriminate the nature of the HexCer molecules incorporated into the UUKV particles, *i.e.*, GlcCer or GalCer (Figs. 1 and S2A and Tables S1 and S2). GlcCer and GalCer are very similar in structure and differ only in the asymmetry of a single hydroxyl group on the fourth carbon (Fig. 1F). It is generally accepted that GlcCer is synthesized by UDP—GlcCer glucosyltransferase (UGCG) in the Golgi, whereas GalCer is generated by UDP—GalCer galactosyltransferase (UGT8) on the luminal side of the ER (Fig. 1B) [21]. The fact that UUKV buds in the Golgi supports the view that GlcCer, rather than GalCer, may be incorporated into the UUKV envelope.

**Fig. 2:**
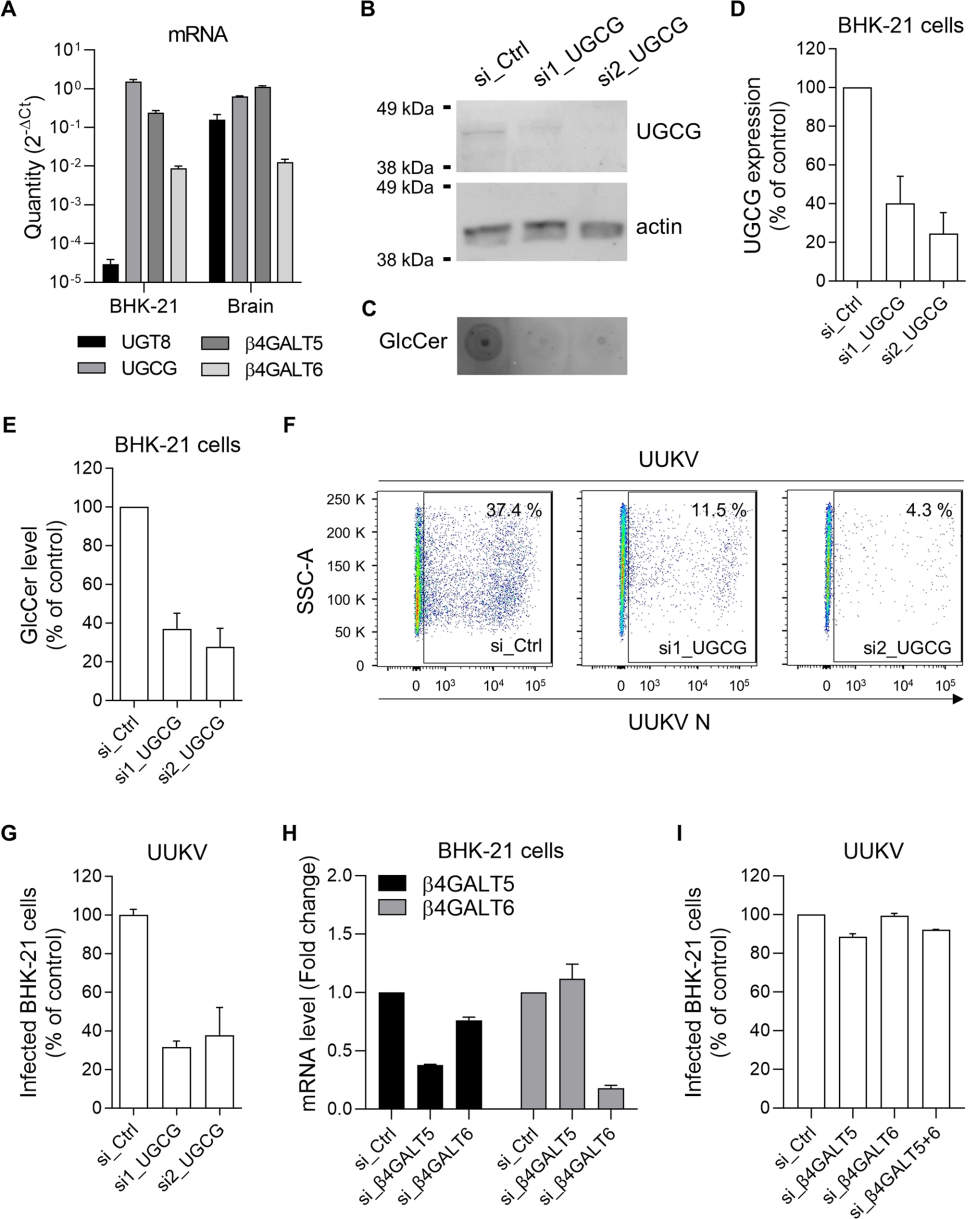
Glucosylceramide (GlcCer) synthase (UGCG) depletion inhibits Uukuniemi virus (UUKV) infection. **A** BHK-21 cells and hamster brain tissues were lyzed and total RNA was extracted before cDNA synthesis. The levels of mRNA encoding UGT8, UGCG, β4Galt5, and β4Galt6 were quantified by RT-qPCR (*n* = 2). **B** UGCG synthase was silenced in BHK-21 cells with two nonoverlapping short interfering RNAs (si1_UGCG and si2_UGCG; 20 nM) and assayed by western blotting 72 h later using the pAb against UGCG from LSBio. SiCtrl (Scrambled_1), negative-control siRNAs. **C** GlcCer levels in BHK-21 cells silenced for UGCG were examined by dot blotting using the same samples as in **B**. **D** The efficiency of UGCG knockdown was semi-quantified under the conditions described in **B**. UGCG protein levels are reported as the percentage of UGCG levels in cells treated with siRNAs against UGCG and normalized to levels of actin and UGCG in BHK-21 cells treated with negative-control siRNAs (si_Ctrl) (*n* = 2). **E** GlcCer levels were semi-quantified based on the dot blots shown in **C** and expressed as a percentage of GlcCer levels in cells treated with siRNAs against GlcCer and normalized to GlcCer levels in BHK-21 cells treated with negative control siRNAs (si_Ctrl, Scrambled_1) (*n* = 6). **F** BHK-21 cells were treated with siRNAs against UGCG (20 nM) for 72 h and then exposed to UUKV (multiplicity of infection (MOI) ~ 0.1). Infection was determined by flow cytometry after immunostaining for the viral nucleoprotein N 24 h post-infection. **G** The values obtained in **F** were normalized to the infection level in samples treated with siRNA controls (si_Ctrl) (*n* = 3). **H** BHK-21 cells were transfected with siRNAs (20 nM) to silence β4Galt5 (si_β4Galt5) or β4Galt6 (si_β4Galt6). Cells were lyzed 72 h post-transfection and total RNA was extracted and purified. The levels of mRNA encoding β4Galt5 or β4Galt6 were quantified by RT-qPCR and expressed as fold change over the values obtained for BHK-21 cells treated with negative control siRNAs (si_Ctrl, Scrambled_2) and set to 1 (*n* = 2). **I** BHK-21 cells silenced for β4Galt5, β4Galt6, or β4Galt5 and β4Galt6 (si_β4Galt5 + 6, 20 nM each) were infected with UUKV at an MOI of 0.1 for 24 h. Infection was then analyzed by flow cytometry as shown in **F**, and the values were normalized to the infection level in samples treated with scrambled siRNAs (*n* = 2)

We further investigated the possibility that GlcCer, not GalCer, is embedded in UUKV particles. GlcCer and GalCer differ not only in their cellular localization but also in their tissue distribution. GlcCer exists in virtually all cell types, while GalCer is predominantly present in cerebral myelin [22, 23]. We, therefore, wondered whether BHK-21 cells, from which UUKV was derived, can produce only GlcCer or both GlcCer and GalCer. For this purpose, we assessed the presence of mRNA encoding UGCG and UGT8 in BHK-21 cells by reverse transcription-quantitative PCR (RT-qPCR) (Fig. 2A). UGCG mRNA was found to be abundant in these cells. In contrast, the mRNA level of UGT8 was five to six logs lower and below the RT-qPCR detection threshold in BHK-21 cells, while it was similar to that of UGCG in baby hamster brain tissue. These data showed that UGT8 was not expressed in BHK-21 cells, indicating that no GalCer could be produced in the cells used to prepare UUKV stocks. From there, we hence focused on UGCG and GlcCer.

To confirm the expression of UGCG and the synthesis of GlcCer in BHK-21 cells, cell lysates were first subjected to SDS-PAGE and western blotting using an antibody targeting UGCG. We found that UGCG was effectively expressed in BHK-21 cells (Fig. 2B). Then, it was possible to detect the presence of GlcCer in BHK-21 cells, as determined by dot blotting with an antibody against GlcCer (Fig. 2C). Altogether, the results demonstrated that BHK-21 cells expressed UGCG and that GlcCer was the prevalent HexCer molecule in the cells used to produce UUKV. GlcCer was evidently the HexCer incorporated into the UUKV particles revealed by our lipidomics analysis.

### UUKV infection relies on GlcCer, not Hex2Cer or GSLs

GlcCer was found to be abundant in UUKV particles. We, therefore, examined whether GlcCer has any functional significance for UUKV infection in tissue culture. We first aimed to disrupt Cer conversion into GlcCer (Fig. 1B) by reducing the expression of UGCG with two in-house-designed, nonoverlapping small interfering RNAs (siRNAs) in parallel experiments. With the application of each siRNA, the level of UGCG in the cultures was reduced by 60–80% (Fig. 2B and D). The silencing of UGCG expression resulted in a decrease in GlcCer synthesis by approximately 60–80% (Fig. 2C and E). We then exposed cells with silenced UGCG to UUKV at a multiplicity of infection (MOI) of 0.1 for 24 h. The sensitivity of these cells to UUKV infection was assessed by flow cytometry analysis after immunostaining for the viral nucleoprotein N (Fig. 2F). Approximately 35–40% of cells transfected with scrambled siRNA were found to be UUKV N-positive in this assay. When UGCG was silenced, UUKV infection was reduced by 60–70% in cells carrying either UGCG siRNA compared with that in the control cells transfected with nontargeted control siRNA (Fig. 2F and G).

Interference with UGCG affects not only GlcCer but also the underlying synthesis of Hex2Cer and complex GSLs. Lactosylceramide (LacCer), a Hex2Cer generated from GlcCer by the LacCer synthases β4GALT5 and β4GALT6 in the Golgi, is generally well accepted to be the starting point for the biosynthesis of all membrane-bound GSLs (Fig. 1B) [24]. RT-qPCR analysis confirmed the expression of β4GALT5 and β4GALT6 mRNA in BHK-21 cells (Fig. 2A). To address whether LacCer and GSLs are required for UUKV infection, we depleted BHK-21 cells of β4GALT5 or β4GALT6 using in-house designed siRNAs in parallel experiments. The level of β4GALT5 and β4GALT6 mRNA in the cultures was reduced by 60–80% as assayed by RT-qPCR (Fig. 2H). Cells treated with β4GALT5 and β4GALT6 siRNAs, independently or in combination, were still efficiently infected by UUKV, at levels similar to those of cells transfected with the control siRNAs (Fig. 2I). Together, these results demonstrated that UUKV infection depends on GlcCer itself, not LacCer or GSLs.

### PPMP and other UGCG inhibitors hinder UUKV infection and spread

**Fig. 3:**
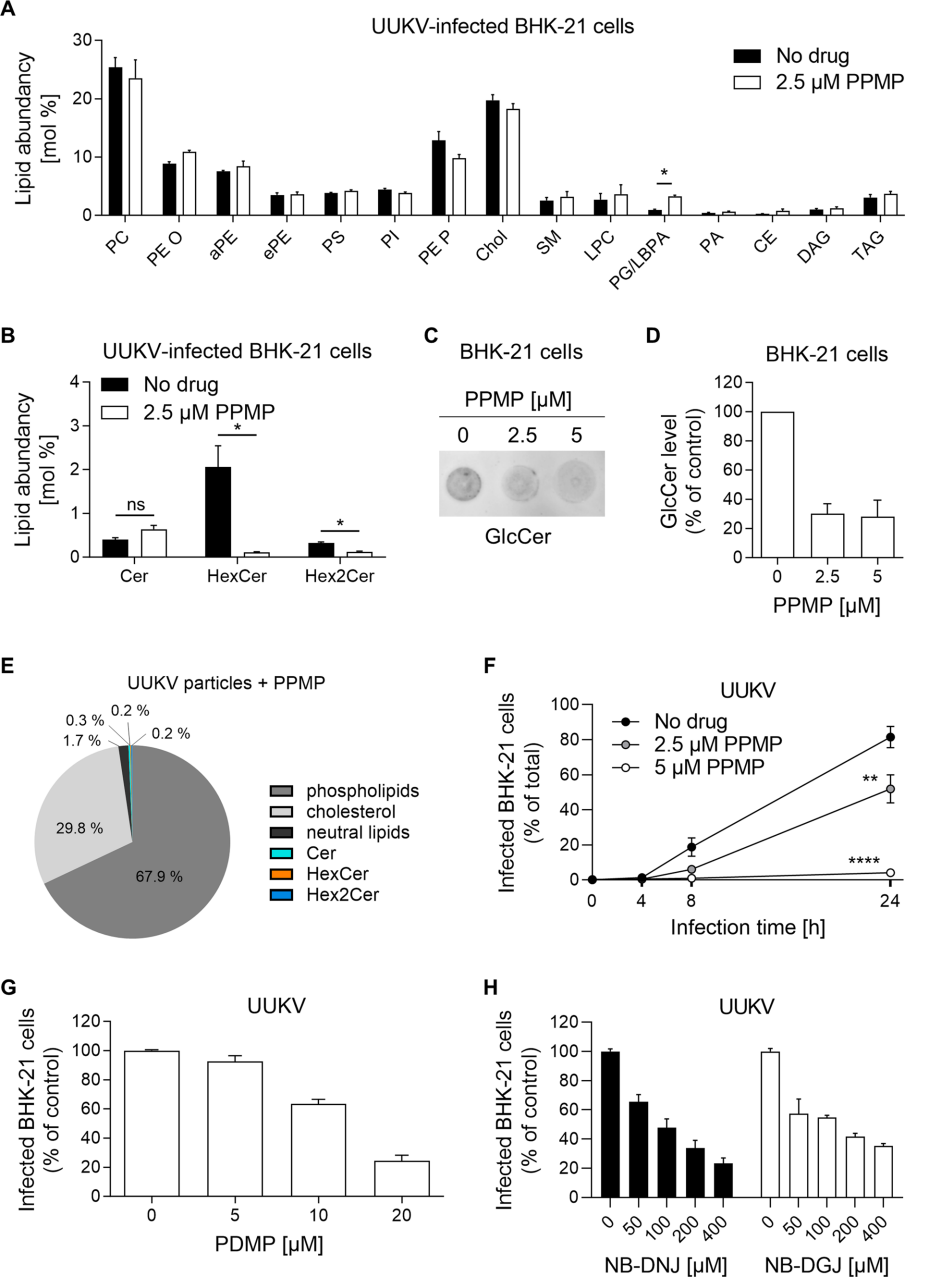
Uukuniemi virus (UUKV) relies on glucosylceramide (GlcCer) in viral particles for infection. **A** BHK-21 cells were pretreated with 2.5 µM of DL-*threo*-1-phenyl-2-palmitoylamino-3-morpholino-1-propanol (PPMP) and then infected with UUKV (multiplicity of infection (MOI) ~ 0.1) in the presence of PPMP, which inhibits UGCG activity. Infected cells were subjected to lipidomics analyses 24 h post-infection. Unpaired t test with Welch correction was applied (*n* = 4). *, *p* < 0.05. **B** Specific analysis of HexCer and Hex2Cer levels in the samples processed as described in **A**. Unpaired t test with Welch correction was applied (*n* = 4). *, *p* < 0.05; ns not significant. **C** GlcCer levels were analyzed in BHK-21 cells after PPMP treatment by dot blotting using an identical number of cells for each sample. **D** GlcCer levels in **C** were semi-quantified and expressed as a percentage of the GlcCer level measured in the absence of PPMP (*n* = 3). **E** 5 × 10^8^ BHK-21 cells in a total of 20 T175 flasks were treated with 2.5 µM PPMP for 16 h before the infection with UUKV. Supernatant from UUKV-infected BHK-21 cells was harvested 24 h post-infection, and UUKV particles were purified before quantitative MS-based lipid analysis. **F** BHK-21 cells were first treated with PPMP for 16 h and then infected with UUKV (MOI ~ 0.5) in the continuous presence of the drug. Infection was detected by flow cytometry up to 24 h later. Note that the standard error of mean (SEMs) of some data series are not visible on the graph. An one-way ANOVA with Dunnett’s multiple comparison test was applied for the time point at 24 hpi (*n* = 3). **, *p* < 0.01; ****p < 0.0001. **G** and **H** BHK-21 cells were pretreated with three other UGCG inhibitors, namely, N-(2-hydroxy-1-(4-morpholinylmethyl)-2-phenylethyl)-decanamide (PDMP), N-Butyldeoxynojirimycin (NB-DNJ), or N-Butyldeoxygalactonojirimycin (NB-DGJ) at the indicated concentrations for 16 h (PDMP) or 24 h (NB-DNJ and NB-DGJ) and were then infected with UUKV (MOI ~ 0.1) in the continuous presence of the drugs. Infection was quantified by flow cytometry after immunostaining for the viral nucleoprotein, and the data were normalized to those in control samples without inhibitor treatment (*n* ≥ 2)

To further investigate the potential involvement of GlcCer in the spread of UUKV in tissue culture, we next assessed the ability of chemical inhibitors of UGCG to prevent UUKV infection. We first treated cells with DL-*threo*-1-phenyl-2-palmitoylamino-3-morpholino-1-propanol (PPMP), a Cer analog [25–27]. We found that the maximal concentration for which PPMP exerted no adverse effects on BHK-21 cell viability after 24-h incubation was 5 µM, as measured with our LDH-based cytotoxicity assay (Figure S3A). BHK-21 cells were, therefore, treated with 2.5 µM of PPMP for 16 h and then exposed to UUKV for a further 24 h before lipid MS analysis. The distribution of the major classes of select lipids, including Cer, was not affected in the UUKV-infected BHK-21 cells that were treated with PPMP (Fig. 3A and B and Table S3). Only the PG/LBPA was enriched, consistent with the accumulation of LBPA in endosomal/lysosomal structures, as has recently been reported for such treatment [28]. Under these conditions, UGCG activity and HexCer synthesis were impeded in UUKV-infected cells. The level of HexCer was less than 0.1% of total lipids (Fig. 3B), *i.e.*, 80–90% lower than the basal level measured in uninfected, untreated cells (Fig. 1A). In agreement with these observations, a dot blot analysis indicated that the GlcCer level was reduced by nearly 80% in uninfected cells after treatment with PPMP (Fig. 3C and D). This reduction was somewhat similar to that observed following the silencing of UGCG (Fig. 2C). Ultimately, treatment of infected cells with PPMP resulted in the production of viral progeny virtually devoid of HexCer (Fig. 3E and Table S4). Taken together, these data demonstrated that PPMP abolished the increase in HexCer in UUKV-infected cells and, in turn, hampered HexCer incorporation into UUKV particles.

To assess the sensitivity of UUKV infection to PPMP, BHK-21 cells were infected at an MOI of 0.1 in the continuous presence of PPMP for up to 24 h. Using our flow cytometry-based infection assay, we observed that PPMP reduced UUKV infection in a dose-dependent manner (Fig. 3F). Of note, PPMP prevented virus propagation as early as 8 h, shortly after the start of the second cycle of viral amplification, which lasts approximately 6 h in BHK-21 cells. To exclude the possibility that this result was cell line-specific, we assessed the effect of PPMP treatment on UUKV infection in the A549 human lung epithelial cell line, which is susceptible to UUKV infection [7]. In the A549 cells, PPMP administered in concentration ranges identical to those administered to BHK-21 cells did not induce cytotoxicity (Figure S3A). Similar to the results obtained with BHK-21 cells, pretreatment of the A549 cells with PPMP blocked UUKV infection (Figure S4).

Through complementary approaches, we examined three other UGCG inhibitors, namely, N-(2-hydroxy-1-(4-morpholinylmethyl)-2-phenylethyl)-decanamide (PDMP), N-butyl-deoxynojirimycin (NB-DNJ), and N-butyl-deoxygalactonojirimycin (NB-DGJ), for their capacity to prevent UUKV infection. PDMP is another Cer analog that is similar to PPMP, while NB-DNJ and NB-DGJ are both glycan analogs that compete with glucose to inhibit UGCG activity [29, 30]. BHK-21 cells were treated with each of these drugs and then infected with UUKV, and the results were similar to those obtained with PPMP. The three drugs each negatively affected UUKV infection in a dose-dependent manner (Fig. 3G and H), while exhibiting no detectable cell toxicity within the range of concentrations applied (Figures S3B and S3C). All inhibitors displayed similar efficiency in preventing UUKV infection, and we used PPMP in the following experiments. Collectively, our results showed that UUKV relies on the enrichment of GlcCer for infection and likely spread.

### UGCG expression increases upon UUKV infection

**Fig. 4:**
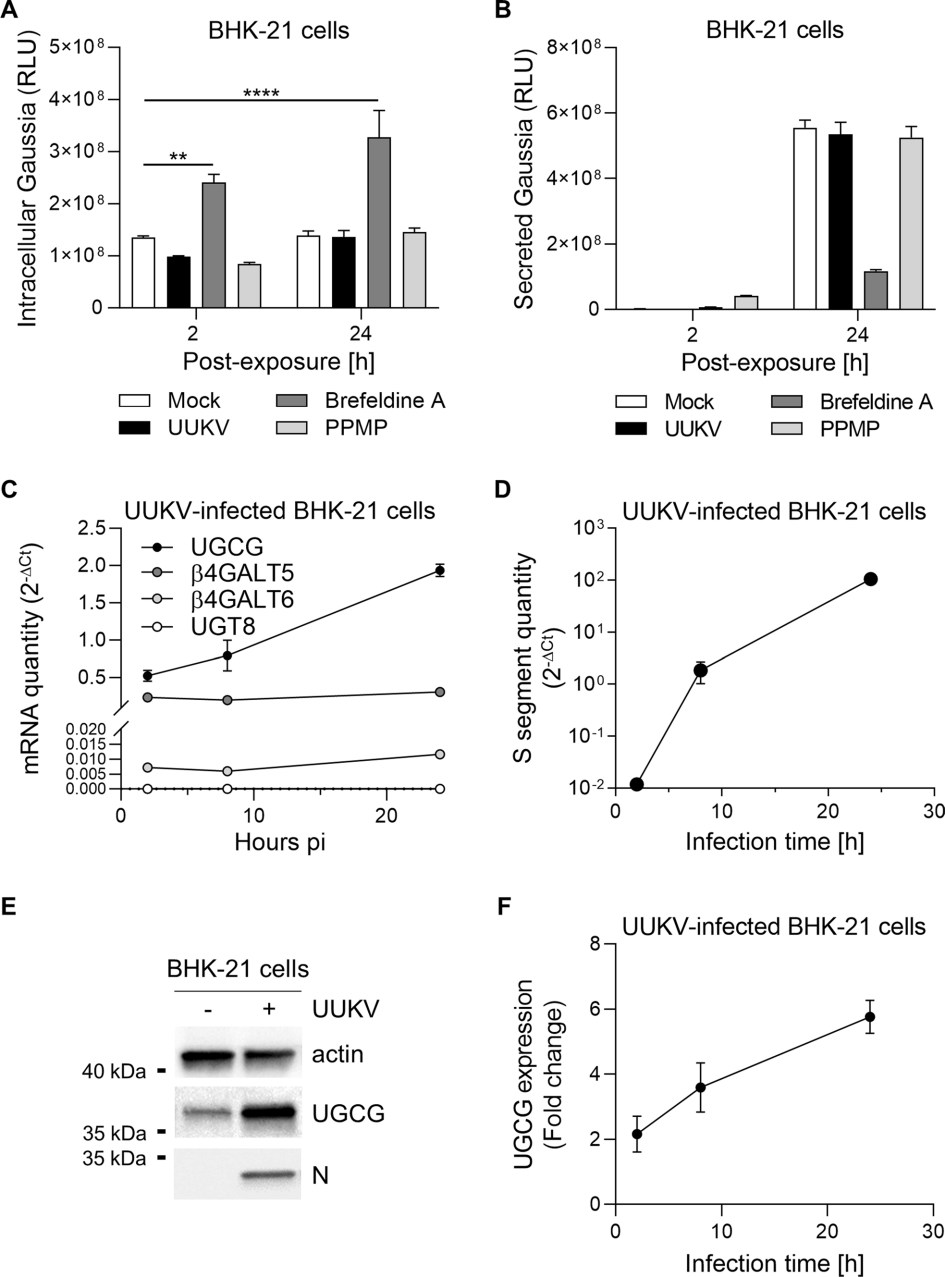
Uukuniemi virus (UUKV) infection induces upregulation of glucosylceramide synthase (UGCG) expression. **A** and **B** BHK-21 cells were transfected with a simian virus 40 (SV40) promoter-driven expression vector encoding Gaussia luciferase (Gluc), which is naturally secreted. One day later, transfected cells were exposed to either UUKV (MOI ~ 1), brefeldin A (1 µM), or PPMP (2.5 µM). Supernatants were collected 24 h post-infection **B**, and cells were lyzed **A**. Luciferase activity was then measured with a Varioskan Lux multimode reader. Two-way ANOVA with Dunnett’s multiple comparison test was applied (*n* = 2). **, *p* < 0.01; ****, *p* < 0.0001. **C** and **D** BHK-21 cells were infected with UUKV (MOI ~ 1) for up to 24 h. Infected cells were collected at different time points and lyzed. Total RNA was then extracted and purified before quantification of mRNAs for UGCG, β4Galt5, β4Galt6, and UGT8 **C** or the S segment of UUKV **D** by RT-qPCR (*n* = 2). Note that the standard error of mean (SEMs) of some data series are not visible on the graph. **E** BHK-21 cells were exposed to UUKV at MOI ~ 0.1 for 24 h and assayed for UGCG and the viral N protein by western blotting using the antibodies 1E5 and 8B11A3, respectively. **F** Shows the semi-quantification of UGCG and N proteins from western blotting analyses as shown in (E) (*n* = 2)

To investigate how UUKV infection resulted in the specific accumulation of GlcCer in BHK-21 cells, we first examined whether UUKV interferes with vesicular trafficking along the secretory pathway. To this end, BHK-21 cells were transiently transfected with a simian virus 40 (SV40) promoter-driven expression vector encoding Gaussia luciferase (Gluc). Gluc has the advantage of being naturally secreted when it is expressed in mammalian cells [31]. Treatment with brefeldin A, a drug that hampers vesicular transport out of the ER, effectively retained Gluc in cells while resulting in a fivefold decrease in Gluc secretion (Fig. 4A and B). Conversely, UUKV had no impact on Gluc release into the culture medium, showing that the secretory pathway remained intact despite infection.

Only the amount of GlcCer, and not Cer and Hex2Cer, was altered upon infection (Fig. 1A). This argues for a scenario in which the accumulation of GlcCer in infected cells is not due to a defect in the conversion of GlcCer to LacCer. To evaluate this possibility, we first monitored UGCG expression after infection. We found that the amount of UGCG mRNA increased strongly (fourfold) over time, correlating with higher viral replication (Fig. 4C and D). The higher mRNA level of UGCG resulted in a sixfold increase in protein expression, as semi-quantified from western blotting (Fig. 4E and F). In contrast, the level of mRNAs encoding the synthases responsible for the conversion of GlcCer to LacCer increased only slightly during the infection period, and not at all for UGT8, the enzyme that converts Cer to GalCer (Fig. 4C). Overall, the results suggested that the enrichment of GlcCer in infected cells was likely due to the overexpression of UGCG.

### GlcCer depletion has no impact on the morphology of UUKV particles

**Fig. 5:**
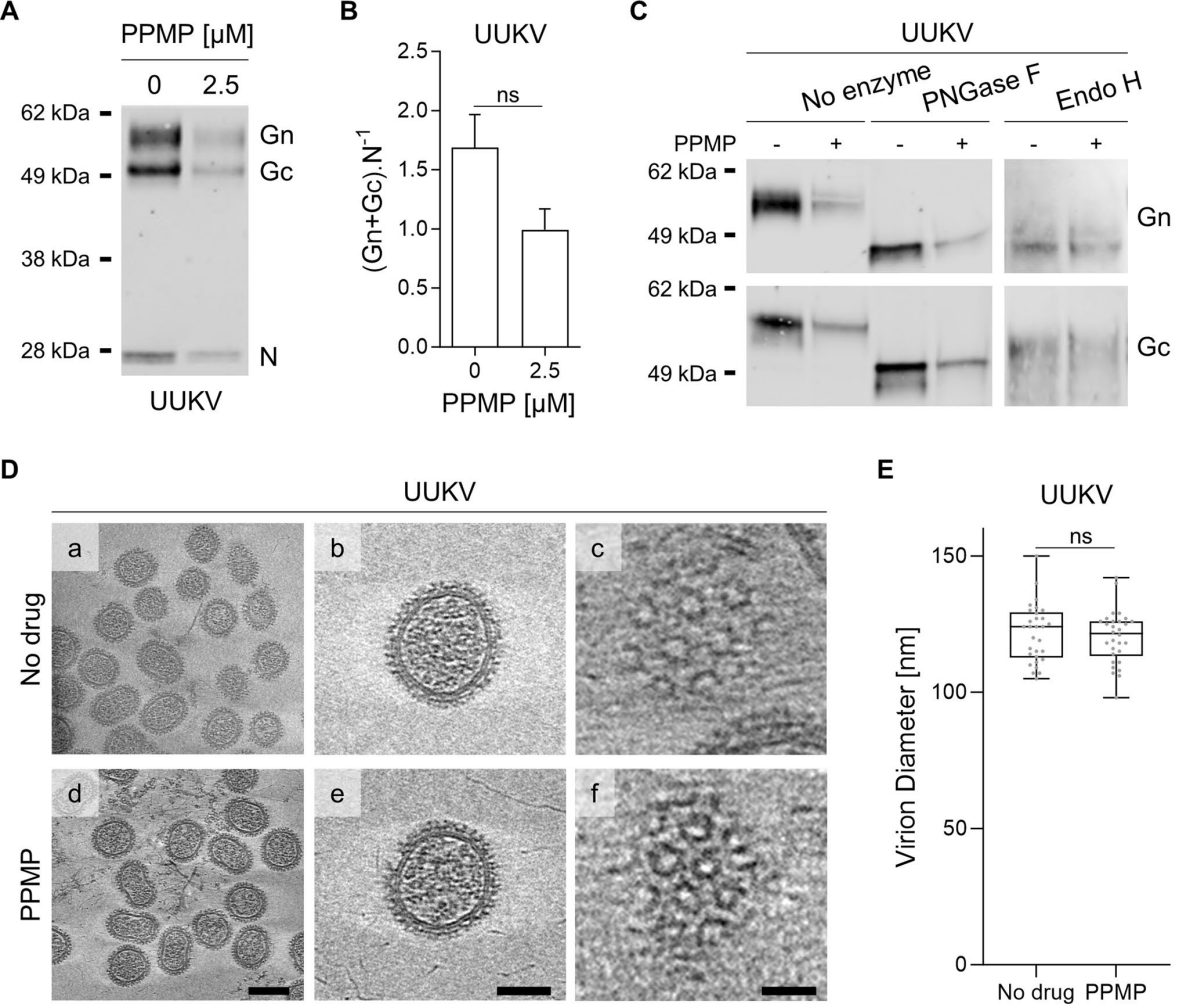
Uukuniemi viruses (UUKVs) with and without glucosylceramide (GlcCer) show no morphological differences. **A** Viral particles produced from BHK-21 cells treated with 2.5 µM of DL-*threo*-phenyl-2-palmitoylamino-3-morpholino-1-propanol (PPMP) were analyzed by SDS-PAGE and western blotting under nonreducing conditions with a polyclonal antibody that recognized UUKV N, Gn, and Gc. **B** UUKV structural proteins in Fig. 5A were semi-quantified. Values are shown as the ratio of the UUKV glycoproteins Gn and Gc level to nucleoprotein N level. Unpaired t test with Welch correction was applied (*n* = 3). ns, not significant. **C** UUKV derived from BHK-21 cells exposed to PPMP was digested with PNGase F (1000 units) or Endo H (2000 units) under reducing conditions for 4 h at 37 ℃. Proteins were separated by SDS-PAGE and analyzed by western blotting with a mAb against either UUKV glycoproteins Gn or Gc. ns, not significant. **D** Cryo-electron tomography of gradient-purified UUKV in the presence or absence of PPMP. (a, d) Slices of tomogram capturing UUKV virions. Scale bars correspond to 100 nm (a, d) and 50 nm (b, e). (c, f) Tomogram slices showing Gn and Gc virion surface arrangement (scale bar: 20 nm). **E** The diameter of UUKV particles produced in the presence or absence of PPMP was measured. Unpaired t test with Welch correction was applied (*n* = 30). *ns* not significant

Host cell receptors have been shown to bind phenuivirus Gn and Gc envelope glycoproteins [3]. We, therefore, evaluated the impact of GlcCer depletion induced by PPMP treatment on the amount of Gn and Gc embedded in viral particles. For this purpose, we determined the ratio of UUKV glycoproteins to N proteins in purified viral particles as analyzed by western blotting (Fig. 5A). This showed no significant difference in the UUKV glycoprotein-to-N ratio whether GlcCer was present in the viral envelope or not (Fig. 5B).

The UUKV glycoproteins Gn and Gc carry several *N*-linked oligosaccharides (four glycosylation sites in each) that contribute to the direct binding of UUKV to C-type lectin receptors [11, 12]. Hence, we analyzed the glycosylation pattern of UUKV Gn and Gc in the viral particles produced in cells depleted from GlcCer because of PPMP treatment. UUKV particles were treated with *N*-glycosidase F (PNGase F) and endoglycosidase H (Endo H) before separation by SDS-PAGE and western blotting (Fig. 5C). Both viral glycoproteins were found to be sensitive to PNGase F and Endo H to a similar extent, whether or not GlcCer was embedded in the viral envelope. The data indicated that *N*-glycan residues in UUKV Gn and Gc do not account for the defective binding of GlcCer-depleted UUKV particles.

Finally, to confirm that PPMP does not impact the overall structural organization of UUKV particles, we imaged viral preparations via cryo-electron tomography (cryo-ET). No apparent difference was observed between the virions produced in the absence or presence of PPMP, indicating that PPMP exerted no impact on virus assembly (Fig. 5D, panels a to f). The typical arrangement of Gn and Gc was visible on the surface of the viral particles regardless of the cell treatment (Fig. 5D, panels c and f). The viral particles were homogenous in shape, and no difference in virion size was detected (Fig. 5E). These findings support the view that each UUKV particle had the same amount of structural proteins whether or not the virus contains GlcCer. Overall, these results suggest that the depletion of GlcCer from the viral envelope does not alter, at least on a large scale, the assembly and structure of UUKV.

### UUKV progeny relies on GlcCer for infectivity

**Fig. 6:**
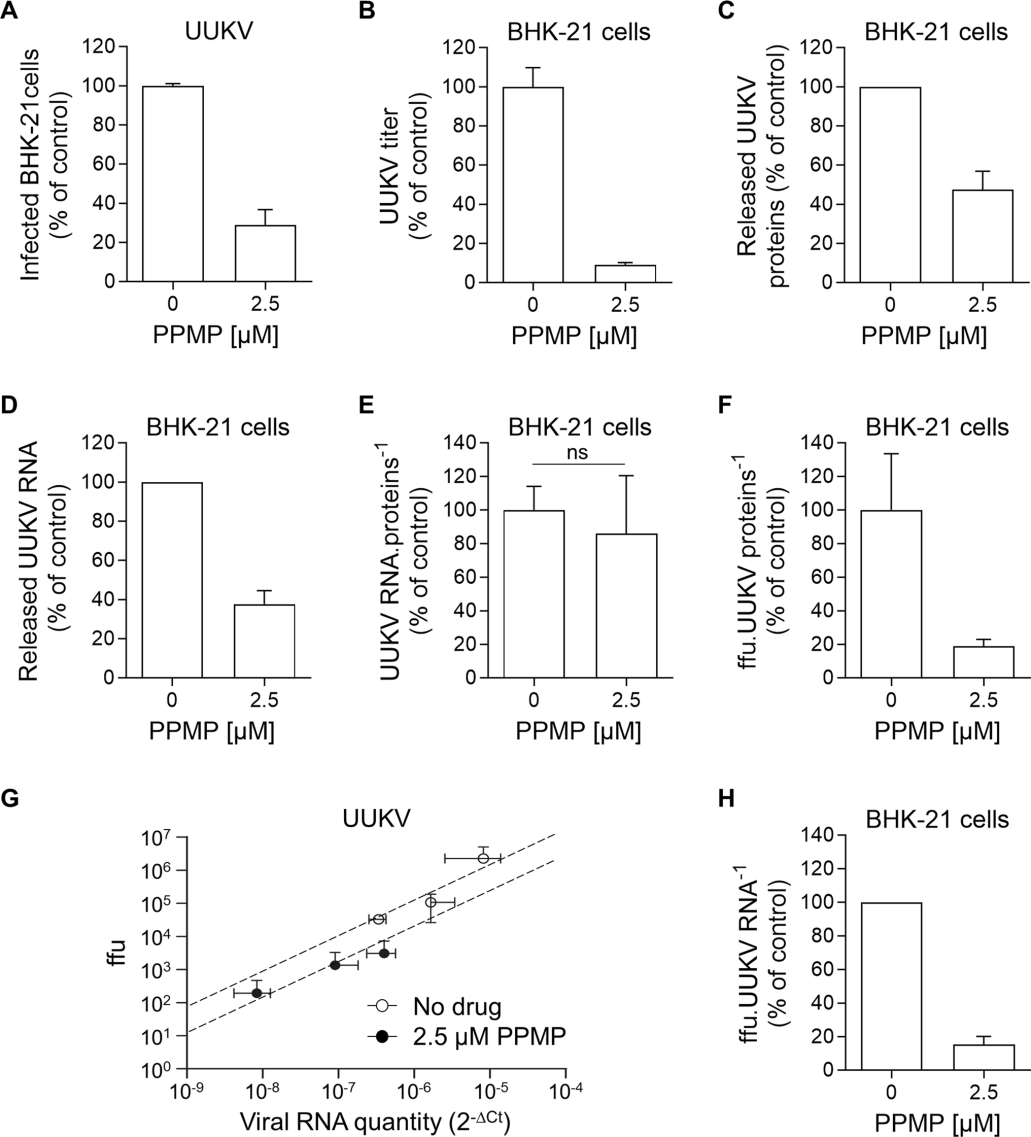
Glucosylceramide (GlcCer) is critical for Uukuniemi virus (UUKV) infectivity. **A** BHK-21 cells were pretreated with DL-*threo*-phenyl-2-palmitoylamino-3-morpholino-1-propanol (PPMP) at the indicated concentrations for 16 h and exposed to UUKV (multiplicity of infection ~ 0.1). The same amount of supernatant of infected cultured cells were harvested 24 h post-infection and then allowed to infect freshly seeded naïve BHK-21 cells for 8 h. The infected cells were immunostained for the detection of UUKV N and analyzed by flow cytometry. The values were normalized to those in control samples where viruses were produced in the absence of the inhibitor (*n* = 3). **B** Infectious UUKV progeny in the supernatant collected from cells cultured in the presence of PPMP was evaluated by focus-forming assay. The data are presented as the percentage of the control samples where viruses were produced in the absence of the inhibitor (*n* = 9). **C** UUKV structural proteins were analyzed by western blotting as described in Fig. 5A and semi-quantified. The data were expressed as a percentage of the level of UUKV N, Gn, and Gc in the supernatant of PPMP-treated and infected BHK-21 cells to the level of N, Gn, and Gc in the supernatant of infected cells that had not been exposed to PPMP (*n* = 6). **D** Viral particles produced from BHK-21 cells in the presence of 2.5 µM of PPMP were analyzed by RT-qPCR using primers that selectively target the RNA segments S, M, and L. The values obtained for each segment were summed, and the data were expressed as a percentage of the RNA content in the supernatant of PPMP-treated and infected BHK-21 cells to the RNA content in the supernatant of infected cells that had not been exposed to PPMP (*n* = 5). **E** UUKV stocks, whether produced in the presence or absence of PPMP, were evaluated for their viral RNA content using RT-qPCR, as detailed in **D**. Their viral structural protein content was also evaluated using western blotting as described in **C**. The ratio of the relative unit of UUKV RNA segments to the relative unit of UUKV structural proteins is presented as the percentage of the value obtained for the control sample without any drug treatment. Ratio paired t test was applied (*n* = 3). *ns* not significant. **F** Same volumes of virus stocks produced in the presence or absence of PPMP were evaluated by both focus-forming assay and western blotting as detailed in **B** and **C**. The ratio of the number of focus-forming units (ffu) to the relative unit of UUKV structural proteins is presented as the percentage of the value obtained for the control sample without any drug treatment (*n* = 6). **G** Identical volumes of UUKV stocks produced in the presence or absence of PPMP were evaluated for their viral RNA content both by foci-forming assay and RT-qPCR as described in **B** and **D**. The values of the three segments were summed, and the relative unit of UUKV RNA segments (2^−∆^^Ct^) was plotted against the number of ffu (*n* = 2). **H** The ratio of the number of ffu to the relative unit of UUKV RNA segments is presented as the percentage of the value obtained for the control sample without any drug treatment (*n* = 5)

Next, we sought to determine the steps in the UUKV cell life cycle that were impaired when UGCG activity was inhibited or GlcCer was depleted. To this end, we first harvested the supernatants from producer BHK-21 cells treated or not with 2.5 µM PPMP. In other words, we produced UUKV particles with or without GlcCer. We then used the same amount of supernatant containing either wild-type viral particles or those lacking GlcCer to infect freshly seeded BHK-21 cells. We observed that the infection of the cells cultured in supernatant from PPMP-treated infected cells was reduced by more than 70% when compared with the infection of cells exposed to the supernatant from mock-treated infected cells (Fig. 6A). We next measured the number of infectious UUKV particles in these same supernatants, *i.e.*, those from BHK-21 cells used to produce UUKV progeny in the presence or absence of PPMP. Performing a standard foci-forming unit titration assay [7], we found that the number of infectious UUKV particles dropped by 90% in the supernatant of the infected cells exposed to PPMP compared with the untreated cells (Fig. 6B).

We subsequently investigated why UUKV stocks, generated in cells depleted of GlcCer using PPMP, have a significantly lower infectious titer. The amount of viral proteins was reduced by 50% in the stocks produced in the presence of PPMP, as determined by western blotting (Figs. 5A and 6C). Identical results were obtained when the viral RNA segments S, M, and L were measured using RT-qPCR (Fig. 6D). The ratio of viral RNAs to structural proteins in virus stocks remained unchanged upon the treatment of producer cells with PPMP (Fig. 6E). These findings are consistent with our electron microscopy images showing that UUKV particles are homogeneous in shape and size whether or not they lack GlcCer (Fig. 5D and E). Altogether, the results suggest that the amount of UUKV genomic RNAs or viral structural proteins can be equated with the number of virions.

The decrease in viral particles released into the supernatant could not be explained by inhibition of the secretory pathway. At this concentration, PPMP had no impact on the secretion of Gluc (Fig. 4A and B). This indicated that virion production is partially inhibited in the presence of PPMP. The decrease in the total number of viral particles by half, however, could not account for the nearly one-log decrease in infectivity of GlcCer-depleted virions (Fig. 6B–D). To clarify whether PPMP exerted an impact on the infectivity of individual viral particles or the release of UUKV particles from producer cells, we determined the ratio of infectious particles to total particles, *i.e.*, the ratio of infectious particles to the total amount of UUKV proteins or genomic RNA. This ratio was reduced by 80% in the medium of PPMP-treated cells using the amount of viral protein equated to the number of virions (Fig. 6F).

To further examine the possibility that individual viral particles are less infectious, we correlated the level of UUKV RNAs in virus stocks, as determined with RT-qPCR, to the number of infectious viral particles. In agreement with our above results, the number of infectious particles was found to be reduced by almost 90% for identical Ct values, *i.e.*, at equivalent RNA levels and hence number of virions, when the virus was produced in the presence of PPMP and devoid of GlcCer (Fig. 6G and H). Overall, the results indicate that the infectivity of UUKV progeny itself was profoundly compromised in the absence of GlcCer and highlighted the importance of GlcCer for the infectivity of viral particles released into the medium and the early steps of UUKV infection.

### GlcCer in the viral envelope promotes UUKV binding to target cells

**Fig. 7:**
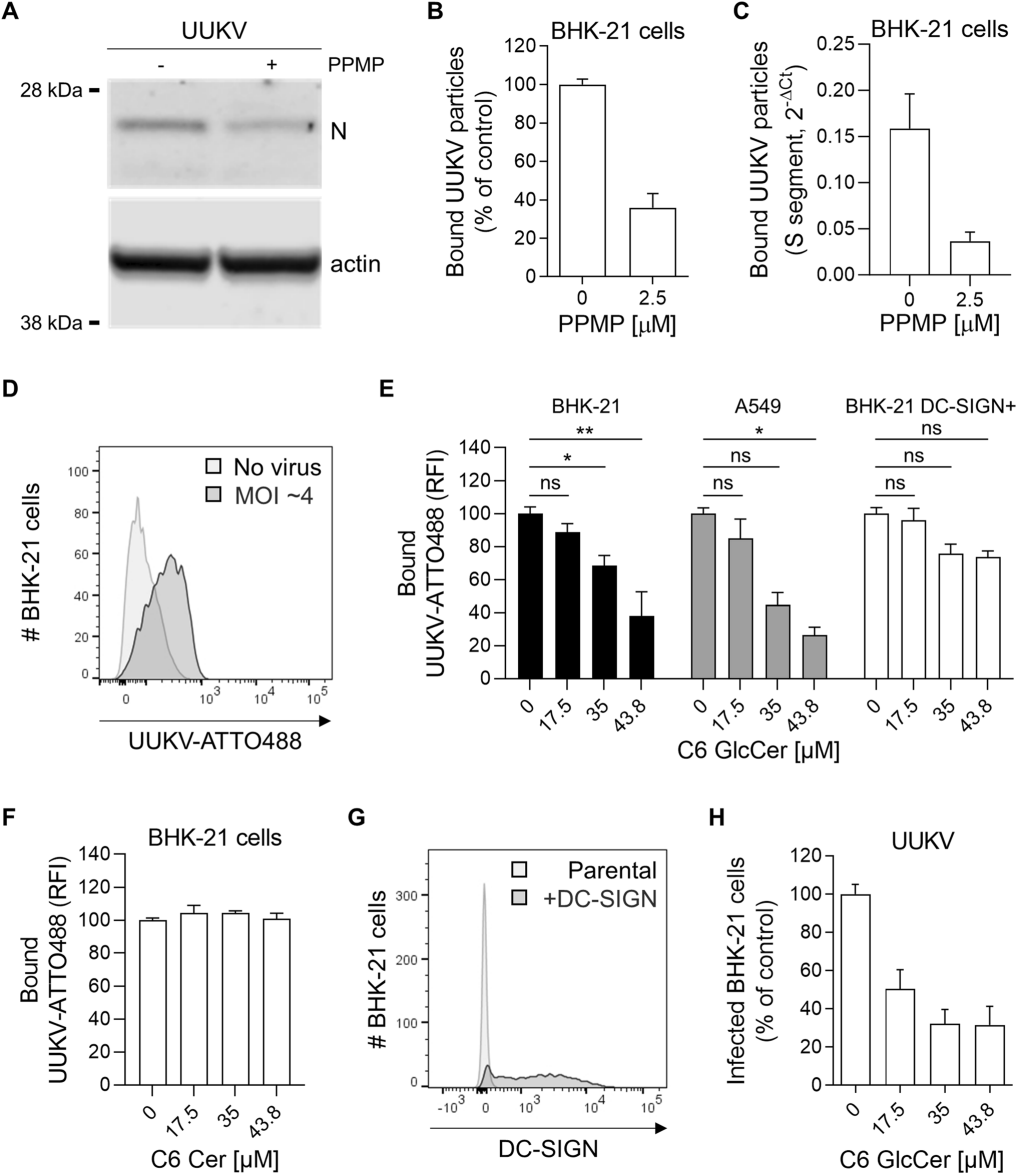
Glucosylceramide (GlcCer) in viral particles promotes Uukuniemi virus (UUKV) binding. **A** UUKV particles derived from BHK-21 cells in the presence of DL-*threo*-phenyl-2-palmitoylamino-3-morpholino-1-propanol (PPMP, 2.5 µM) were bound to freshly seeded naïve BHK-21 cells for 2 h on ice before fixation and western blot analysis with an antibody recognizing the UUKV N protein. **B** N was semi-quantified from the cells described in **A**, and the value is presented as a percentage of the N level measured in the sample corresponding to virus binding in the absence of PPMP (*n* = 8). **C** Alternatively, UUKV particles produced in the presence or the absence of PPMP were allowed to bind to BHK-21 cells for 2 h on ice, and binding was assessed by measuring the BHK-21 cell-associated S viral segment by RT-qPCR (*n* = 6). **D** Fluorescently labeled UUKV particles (UUKV-ATTO488) were bound to BHK-21 cells [multiplicity of infection (MOI) ~ 4] on ice for 1 h, and viral binding was evaluated by flow cytometry analysis. **E** and **F** BHK-21 cells, A549 human lung epithelial cells, and BHK-21 cells expressing the UUKV receptor DC-SIGN (BHK-21 DC-SIGN +) were preincubated with varying amounts of soluble C6-GlcCer **E** or C6-Cer **F** for 2 h and then exposed to UUKV-ATTO488 (MOI ~ 4) on ice for 1 h. Virus binding was measured by flow cytometry, and the data were normalized to those in control samples processed in the absence of soluble C6-GlcCer or C6-Cer. An one-way ANOVA with Dunnett’s multiple comparison test was applied (*n* ≥ 3). *, *p* < 0.05; **, *p* < 0.01; ns, not significant; RFI, relative fluorescence intensity. **G** BHK-21 cells were transduced with a retroviral vector system to express DC-SIGN (BHK-21 DC-SIGN +). DC-SIGN expression was measured by flow cytometry analysis using phycoerythrin-conjugated anti-DC-SIGN mAb. **H** Soluble C6-GlcCer was allowed to bind BHK-21 cells on ice for 2 h before exposure to UUKV for 1 h (MOI ~ 0.5). After virus binding on ice, unbound UUKV particles were washed away, and the cells were incubated at 37 ℃ for 8 h. Infection was quantified by flow cytometry after immunostaining for UUKV N protein. Values are presented as the percentage of the control sample without prebinding of soluble C6-GlcCer (*n* = 4)

Our results suggested that UUKV relies on GlcCer for infectious entry. To confirm this possibility, we evaluated the role played by GlcCer in virus binding to BHK-21 cells. To this end, the amount of input virus was normalized based on the UUKV N protein level in the virus stocks as measured by western blotting and not the MOI. The N level reflects the total number of viral particles, in contrast to the MOI, which reveals only infectious virus levels. UUKV particles were produced in the presence or absence of PPMP for 24 h. Fresh, naïve BHK-21 cells were then exposed to virions on ice for 2 h, extensively washed, and subjected to SDS-PAGE and western blot analysis (Fig. 7A). Strikingly, the amount of UUKV nucleoprotein N bound to the BHK-21 cells was reduced by 60–70% when the viral particle envelopes lacked GlcCer (Fig. 7A and B). Similar observations were made when virus binding was analyzed by RT-qPCR (Fig. 7C).

To further investigate the possibility that GlcCer in the viral particles directly promotes the binding of viral particles to host cells, we subjected ATTO488-conjugated UUKV (UUKV-ATTO488) to a flow cytometry-based binding competition assay (Fig. 7D) [12, 32]. The binding of fluorescently labeled UUKV particles to BHK-21 and A549 cells at 4 °C was found to be inhibited by the prebinding of soluble C6-GlcCer, but not C6-Cer and the effect was dose-dependent (Fig. 7E and F). In contrast, DC-SIGN expression in BHK-21 cells, which do not endogenously express this lectin (Fig. 7G), largely preserved UUKV binding in the presence of prebound C6-GlcCer (Fig. 7E). This finding indicated that UUKV binding can occur through interactions between DC-SIGN and mannose residues in the Gn and Gc glycoproteins or GlcCer present in the viral envelope and an unknown receptor in BHK-21 cells. In addition, binding competition between soluble C6-GlcCer and UUKV before incubation at 37 °C significantly reduced viral infection (Fig. 7H). Taken together, these experiments indicated that UUKV binding to BHK-21 and A549 cells is mediated through GlcCer in the viral envelope and that binding most likely involves specific attachment factors or receptors but not DC-SIGN.

### GlcCer in target cells is not essential for UUKV membrane fusion

**Fig. 8:**
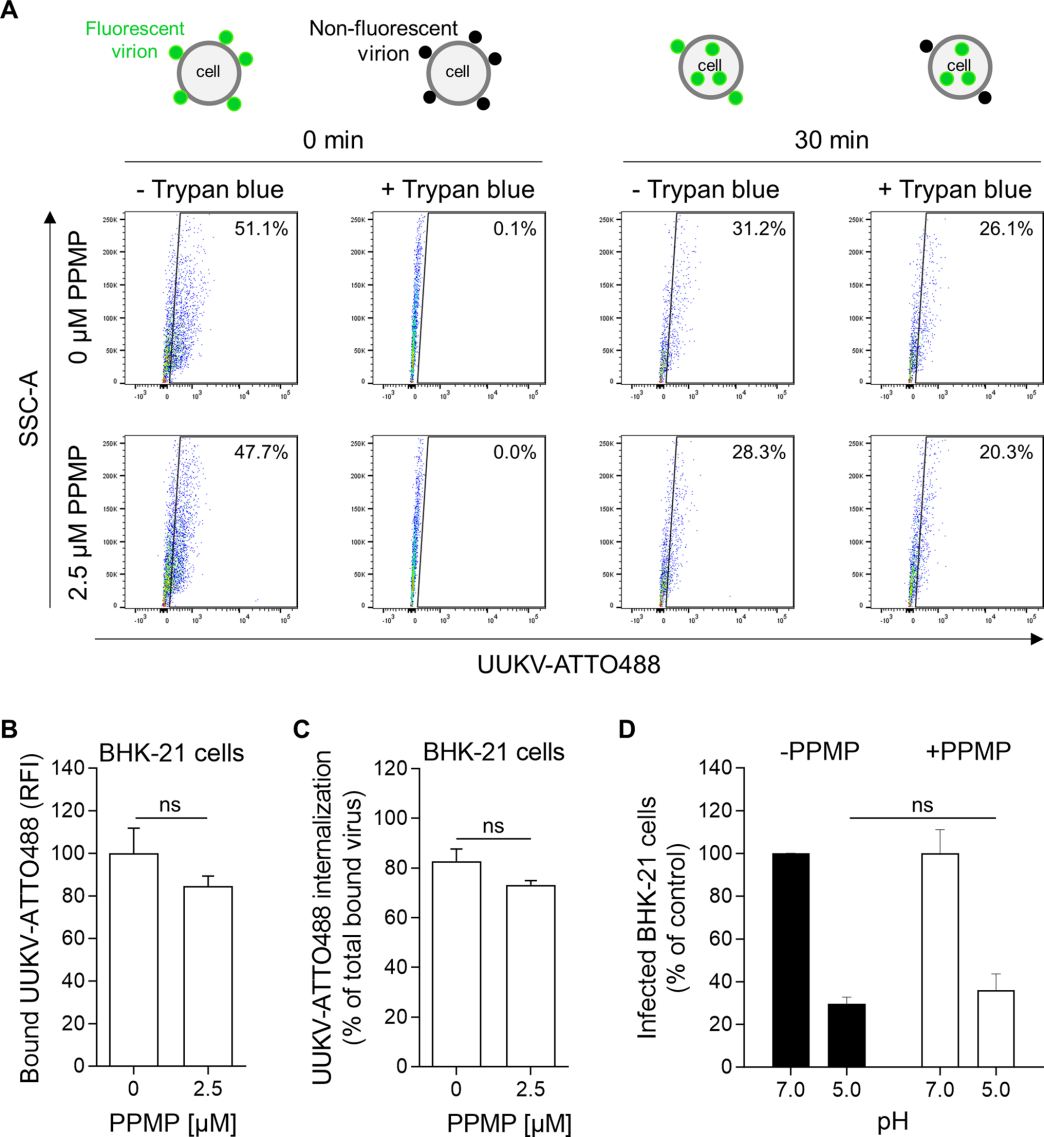
GlcCer in target cells is not essential for Uukuniemi virus (UUKV) entry and fusion. **A**–**D** BHK-21 cells were treated with PPMP for 16 h at 37 ℃ before UUKV binding. **A** Cells were exposed to UUKV-ATTO488 (MOI ~ 4) in suspension at 4 ℃ and rapidly warmed to 37 ℃ to allow virus uptake for 30 min. Endocytic internalization of virions was determined by flow cytometry after trypan blue treatment. **B** UUKV-ATTO488 (MOI ~ 4) was allowed to bind to cells on ice before fixation and analysis by flow cytometry. Virus binding is expressed as the relative fluorescence intensity associated with the cells, as measured by flow cytometry. Unpaired t test with Welch correction was applied (*n* = 2). ns, not significant. **C** Internalization is given as the percentage of fluorescence quantified in samples treated with trypan blue as compared to that in untreated samples. The fluorescence signal measured in cells not exposed to UUKV-ATTO488 was considered the background signal and subtracted from the other values. Unpaired t test with Welch correction was applied (*n* = 2). ns, not significant. **D** UUKV was bound at MOI ~ 5 to cells on ice. Subsequently, cells were washed and subjected to pH ~ 5.0 at 37 ℃ for 90 s to trigger the virus fusion at the plasma membrane. Infected cells were then incubated for 7 h at 37 ℃ in the presence of 50 mM NH_4_Cl to prevent viral penetration from endosomes, and thereby only monitor the release of viral genomes from the plasma membrane. Infection was quantified by flow cytometry, and the data were normalized to those from samples where cells were not treated with PPMP. Unpaired *t* test with Welch correction was applied (*n* = 2). *ns* not significant

To evaluate the importance of GlcCer in target cell membranes for infectious entry of incoming viruses, we analyzed each step of the UUKV entry process in cells pretreated with PPMP. We did not observe a significant difference in the binding of UUKV-ATTO488 to BHK-21 cells, whether they were depleted of GlcCer or not (Fig. 8A and B). To monitor virus endocytosis, the binding of UUKV-ATTO488 to BHK-21 cells was synchronized on ice, and then the cells were rapidly warmed to allow the internalization of viral particles. To discriminate between surface-bound and internalized viral particles, cells were subjected to trypan blue before flow cytometry analysis [12]. Trypan blue is a membrane-impermeable dye that quenches the fluorescence emitted by surface-exposed UUKV-ATTO488, while internalized viruses remain fluorescent. When the amount of trypan blue-resistant fluorescence of cell-associated UUKV-ATTO488 was analyzed 30 min post-warming, no significant difference was observed between cells pretreated with PPMP or untreated (Fig. 8A and C).

The fusion of the viral envelope with cellular membranes is the final step in the entry program of enveloped viruses into the host cells. To assess the importance of GlcCer in this crucial step of the viral entry process, we forced the fusion of UUKV with the plasma membrane of cells pretreated with PPMP, as previously described [7]. In this approach, we rely on the ability of the virus to enter host cells through the plasma membrane and bypass the need for endocytosis during productive infection. Briefly, UUKV was allowed to bind to cells on ice. Then, the temperature was rapidly shifted to 37 ℃ for 1.5 min in buffers with different pHs, and NH_4_Cl-containing medium at neutral pH was added for the remaining infection period. NH_4_Cl is a lysosomotropic weak base that neutralizes vacuolar pH and prevents infection via endosomes. The bypass resulted in efficient infection regardless of the presence or absence of GlcCer (Fig. 8D), conclusively demonstrating that viral membrane fusion can occur in the absence of GlcCer. Altogether, the results indicated that GlcCer in target cells was not essential for the entry and fusion of incoming UUKV particles.

### GlcCer is necessary for infectious entry of phenuiviruses and other bunyaviruses

**Fig. 9:**
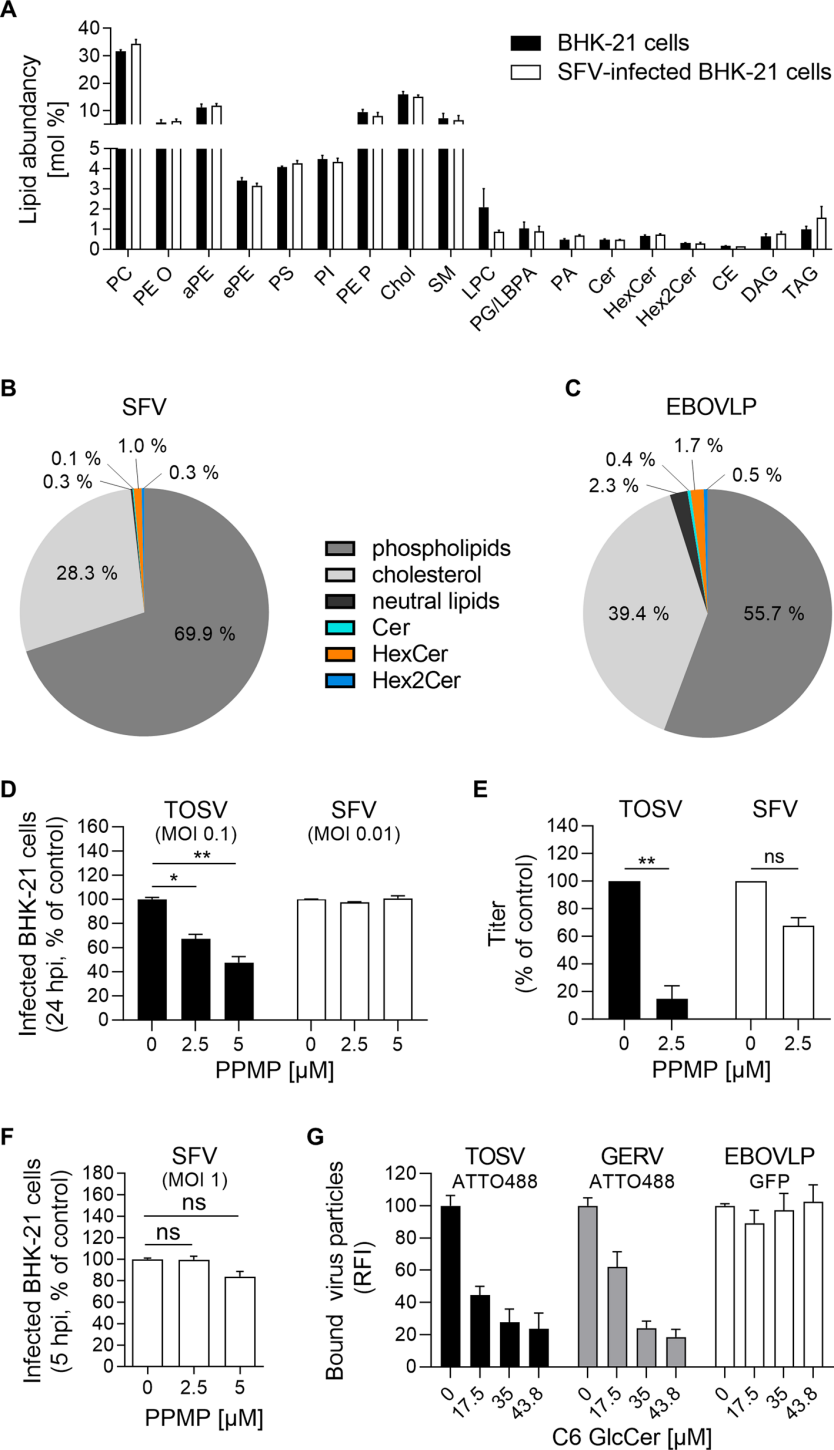
Glucosylceramide (GlcCer) is important for the attachment of viruses that bud from the Golgi. **A** BHK-21 cells were infected with Semliki forest virus (SFV) at a multiplicity of infection (MOI) of 0.01 and subjected to lipidomic analyses 14 h post-infection (*n* = 3). **B** SFV particles were harvested 24 h post-infection and purified from the supernatant of infected cells and included in the analysis (*n* = 3). **C** Ebola virus-like particles (EBOVLPs) were produced from HEK293T cells and purified before the lipidomic analysis with MS (*n* = 3). **B**, **C** Phospholipids include all glycerophospholipids, sphingomyelin (SM), and lysophosphatidylcholine (LPC). The neutral lipids were cholesteryl ester (CE), diacylglycerol (DAG), and triacylglycerol (TAG). **D** BHK-21 cells were first treated with DL-*threo*-phenyl-2-palmitoylamino-3-morpholino-1-propanol (PPMP) at the indicated concentrations for 16 h and then infected with either TOSV (MOI ~ 0.1) or SFV (MOI ~ 0.01) in the continuous presence of PPMP. Infection was measured by flow cytometry 24 h later. An one-way ANOVA with Dunnett’s multiple comparison test was applied (n ≥ 2). *, *p* < 0.05; **, *p* < 0.01. **E** Infectious progeny of TOSV and SFV produced by BHK-21 cells in the presence of PPMP for 24 h was evaluated by focus-forming assay. The data are presented as the percentage of the control samples where viruses were produced in the absence of an inhibitor. Ratio paired t test was applied (*n* = 3). **, *p* < 0.01; ns, not significant. **F** BHK-21 cells were pretreated with PPMP at the indicated concentrations for 16 h and exposed to SFV (MOI ~ 1) for 5 h. Infected cells were immunostained for the SFV glycoprotein E2 and analyzed by flow cytometry. An one-way ANOVA with Dunnett’s multiple comparison test was applied (*n* = 3). *ns* not significant. **G** Cells were preincubated with the indicated amounts of soluble C6-GlcCer for 2 h on ice and then exposed to ATTO488-labeled TOSV (TOSV-ATTO488) and ATTO488-labeled Germiston virus (GERV-ATTO488), and EBOVLPs containing green fluorescent protein (EBOVLP-GFP) on ice for 1 h. TOSV-ATTO488 and GERV-ATTO488 were used to infect cells at MOIs of 4 and 15, respectively. Approximately 500 ng of EBOVLP-GFP total protein was used to bind to 1 × 10^5^ cells. Virus binding was measured by flow cytometry, and the data were normalized to those in control samples processed in the absence of soluble C6-GlcCer (*n* ≥ 3). *RFI* relative fluorescence intensity

Next, we examined whether other viruses depend on GlcCer to bind to target cells. First, we analyzed BHK-21 cells via our lipidomic with MS approach after the cells were infected with Semliki forest virus (SFV), an unrelated virus from the *Togaviridae* family that assembles at the plasma membrane [33, 82]. SFV did not alter the distribution of Cer, HexCer, or Hex2Cer in infected cells (Fig. 9A and Table S5), suggesting that infection by SFV does not interfere with the GSL synthesis pathway. Next, SFV particles produced by these cells were subjected to lipid MS analysis after purification, and the results also did not indicate increased GlcCer incorporation into the virions (Figs. 9B and S5, and Table S2). Identical results were obtained with Ebola virus-like particles (EBOVLPs), which also bud from the plasma membrane (Fig. 9C) [34]. The proportion of HexCer did not exceed 1.0–1.7% of the SFV and EBOVLP envelope, in contrast, 3.6% of the UUKV envelope consists of HexCer (Fig. 1C).

To evaluate the functional importance of GlcCer to human pathogenic bunyavirus infection, we extended our investigation to include TOSV. TOSV is the primary cause of arboviral diseases in humans in southern Europe during the summer season [35–37]. Similar to other bunyaviruses, TOSV buds into the Golgi network. BHK-21 cells were infected with TOSV in the presence of PPMP. We observed that the TOSV infectivity decreased with increasing concentrations of PPMP (Fig. 9D). In addition, the release of infectious progeny was severely inhibited in the presence of PPMP (Fig. 9E). In contrast, GlcCer depletion by PPMP had exerted no adverse effect on SFV infection, irrespective of the MOI, or on the subsequent production of infectious viral particles (Fig. 9D–F). Finally, we assessed the capacity of soluble C6-GlcCer to inhibit TOSV binding. For this assay, purified stocks of TOSV were labeled with the fluorescent dye ATTO488 [38], and then, the fluorescent virus (TOSV-ATTO488) was allowed to bind BHK-21 cells in the presence of increasing soluble C6-GlcCer concentrations on ice for 1 h. Soluble C6-GlcCer abolished TOSV binding (Fig. 9G). Similar results were obtained when Germiston virus (GERV) was tested in a manner identical to TOSV (Fig. 9G). GERV is a bunyavirus of the family *Peribunyaviridae* and is thus related to phenuiviruses [8]. Similar to phenuiviruses and other bunyaviruses, peribunyaviruses bud and assemble in the Golgi network [39–41]. As expected, EBOVLP binding was not affected by soluble C6-GlcCer (Fig. 9G).

Collectively, the results suggested that viruses incorporate the glycolipid GlcCer when leaving the cell from the Golgi network but not from the plasma membrane, consistent with the preferential location of GlcCer and its transformation from Cer in this organelle. Overall, our study demonstrated that the glycolipid GlcCer mediates virus attachment to host cells, presumably through specific interactions with cellular receptors.

## Discussion

The lipid composition of enveloped viruses plays a major role in the structural organization of virions, which in turn contribute to virus binding and membrane fusion for the efficient release of viral genomes into cells. In this study, we focused on mammalian host cells and evaluated the impact of a bunyavirus, *i.e.*, UUKV, on the lipidome of infected cells and determined the lipid composition of the envelope of newly produced viral particles. Except for HexCer, UUKV did not alter the lipid composition of infected cells. The strong enrichment of Chol, SM, and PE P in the viral envelope supports a budding of viral particles from post ER/post *cis* Golgi compartments. This is consistent with the accumulation of the UUKV glycoproteins Gn and Gc more in *trans* in the Golgi network, from where virions bud [5, 42].

Our results clearly show that UUKV incorporates the glycolipid GlcCer in its envelope to attach to host cells. We also found that GlcCer promotes virus binding and infection by TOSV. These data are in agreement with studies showing that the infectious entry of the bunyavirus DABV relies on UGCG [43], the enzyme responsible for the synthesis of GlcCer from Cer in the Golgi [44]. A role for UGCG has also been proposed in infection by the unrelated influenza virus, Sindbis virus, and severe acute respiratory syndrome coronavirus 2, SARS-CoV-2 [45–47]. Although the GlcCer content of DABV and other bunyaviruses as well as SARS-CoV-2 remained to be determined experimentally, many viruses that assemble in the Golgi apparatus can likely use GlcCer to infect host cells. Most bunyaviruses are arboviruses, and whether GlcCer is also present in the envelope of arthropod cell-derived bunyaviruses and has a role in transmission to vertebrate hosts remains an important objective.

One bunyavirus, UUKV, was used to further analyze the role of glycolipids in the infection process. GlcCer was found to be enriched in infected cells. The reason is probably that UGCG was overexpressed upon infection compared with β4Galt5 and β4Galt6, the two LacCer synthases responsible for the conversion of GlcCer to LacCer. In this model, the excess GlcCer produced by larger amounts of UGCG would not be fully processed by LacCer synthases in the Golgi, and GlcCer would accumulate. UUKV, like other bunyaviruses, buds into the Golgi, and one or more of the five UUKV proteins may also, directly or indirectly, regulate host cellular proteins that govern the GSL synthesis pathway. Whether GlcCer accumulation creates a lipid cluster in the Golgi that provides a platform for virus budding and assembly has yet to be studied. Further functional investigations will also be needed to understand the detailed mechanisms of the specific accumulation of GlcCer during UUKV infection.

Our lipidomic MS approach showed that HexCer not only accumulates in the infected cells but is also incorporated into the envelope of UUKV viral particles. The structures of GlcCer and GalCer are very similar, and our lipidomic MS analysis did not allow discrimination between the two types of HexCer molecules. However, the conversion from Cer to GalCer is ensured by UGT8 in the ER [21], incompatible with UUKV budding in the Golgi, and UGT8 was not expressed in the cells used to produce UUKV. In contrast, Cer is primarily converted to GlcCer by UGCG in the Golgi of most cell types [18], and UGCG was present in our UUKV-producing cells. Consistent with this, our subsequent analysis confirmed that GlcCer and not GalCer plays a role in UUKV infection.

Our results indicate that GlcCer was also embedded in both TOSV and GERV particles. In contrast, SFV and EBOVLPs assemble at the plasma membrane, and our analysis showed no significant level of GlcCer level in their respective viral envelope. The lipid composition of other viruses that bud on the cell surface [33, 48], such as vesicular stomatitis virus (VSV), has been analyzed, and GlcCer was not found in the VSV envelope [49]. Moreover, an excessive level of GlcCer is detrimental to the cell cycle of influenza A virus [46, 50]. In the same vein, dengue virus, a flavivirus that buds into the ER, downregulates ceramides [51]. Although more viruses need to be analyzed to support these findings, GlcCer is likely incorporated only into the envelope of viruses that assemble in the Golgi.

Our data clearly showed a biological function for GlcCer in the viral envelope. The glycolipid contributed to both viral binding and infection. Soluble C6-GlcCer prevented UUKV, TOSV, and GERV attachment, but did not affect EBOVLP binding, consistent with the virtual absence of GlcCer in the EBOVLP envelope. To our knowledge, so far only PS has been described to be associated with the viral envelope to facilitate the binding of different viruses, namely, Chikungunya virus, dengue virus, vaccinia virus, EBOV, and HIV [52–56]. However, PS significantly differs from GlcCer in several respects. For example, in contrast to GlcCer, PS is a negatively charged lipid abundant and preferentially exposed on the inner leaflet of the plasma membrane. In contrast, the GlcCer pathway appears to be finely and temporally regulated during UUKV infection; however, the details of the mechanisms remain to be discovered.

Our findings contrast with recent reports on DABV and HRTV, for which GlcCer was proposed to facilitate viral entry and membrane fusion by being recognized in the endosomal membranes of target cells via a lipid-binding pocket in the viral Gc protein [43, 57]. This virus may use GlcCer differently. A lipid-binding pocket in Gc has been described as playing a role in RVFV fusion following its binding to cholesterol [58], although this lipid is rare in the late endosomes from which most bunyaviruses fuse and enter the cytosol [3]. However, we cannot exclude that GlcCer by itself in the viral envelope also mediates HRTV binding to host cells. First, GlcCer is not localized in late endosomes [21, 24]. Then, the investigation with HRTV was mainly based on the use of pseudotyped particles that bud from the plasma membrane, whereas GlcCer is located in the Golgi [21, 24]. Authentic viruses were used, but only at late stages, when several infection cycles had occurred. UUKV allowed the lipidomic characterization of authentic virions and accurate analysis of virus entry within a few minutes after binding.

Our results suggested that a high level of GlcCer in the viral envelope promotes UUKV binding. Despite the Gn—Gc lattice covering virions, GlcCer is likely accessible in the viral envelope to one or more receptors on the host cell surface, reminiscent of PS and flavivirus binding. The phospholipid receptor CD300a recognizes PS associated with flavivirus particles underneath the dense lattice of viral envelope glycoproteins [59]. With an ectodomain often exceeding 10–15 nm in length, lectins, among other cellular factors, represent interesting candidates to examine for their ability to reach and recognize GlcCer through the Gn—Gc coat, which is approximately 10 nm thick. Several lectins act as an attachment factor or receptor for bunyaviruses, including UUKV, TOSV, and GERV [11, 12, 60, 61].

Specifically, receptors may recognize the lipid core of GlcCer or the GlcCer molecules in the viral envelope may form larger clusters with other lipids in the plasma membrane of target cells, bringing the viruses close to receptor complexes. The fact that soluble ceramide does not interfere with UUKV binding suggests an alternative scenario, in which the glucose moiety on ceramide is involved in virus—host cell receptor interactions. Macrophage-inducible C-type lectin (Mincle) recognizes glucose residues and GlcCer, among other glycolipids [62]. In addition, this lectin has recently been shown to bind RVFV and La Crosse virus, a peribunyavirus similar to GERV [60, 61]. Further investigations will be needed, not only to determine whether Mincle is an attachment factor or receptor for UUKV and other viruses carrying GlcCer molecules in their envelope but also to identify the receptors that can capture enveloped viruses through GlcCer.

Significant efforts have been made to identify virus receptors. However, although thousands of viruses have been sequenced, only a few receptors have been described to date. Most researchers have focused on interactions between host cell surface factors and viral envelope glycoproteins or the carbohydrates they carry [63]. Lipids in viruses are difficult to investigate and only a handful of viral lipidomes have thus far been reported. Thus, the role of viral envelope lipids in infectious entry remains largely enigmatic. Our lipidomic analysis with MS of UUKV expanded the known virus lipidomes. By acting as a crucial molecular compound in the viral envelope that promotes virus binding to cells, the glycolipid GlcCer plays a critical role in UUKV—receptor interactions and viral tropism. In other words, our data point to a new type of virus—receptor interaction: the interaction between glycolipids in the viral envelope and receptors on the host cell surface. We propose that, with the ability to incorporate glycolipids into their envelope, viruses can modulate tropism and increase interactions with host cells via glycan residues in their envelope, not solely through their viral glycoproteins. The results presented here offer new perspectives based on this novel virus-binding modality for future receptor identification.

## Methods

### Cells

All products used for cell culture were obtained from Thermo Fisher Scientific or Merck. BHK-21 cells are baby hamster kidney cells, derived from the Syrian golden hamster, and were grown in Glasgow’s minimal essential medium (GMEM) supplemented with 10% tryptose phosphate broth (TPB) and 5% fetal bovine serum (FBS). BHK-21 cells that stably express the human C-type lectin DC-SIGN were obtained by transduction with a TRIPΔU3 lentiviral vector encoding DC-SIGN as previously reported [64]. Human A549 lung and HEK293T kidney cells were cultured in Dulbecco’s Modified Eagle’s Medium (DMEM) supplemented with 10% FBS. The medium for culturing A549 cells was complemented with 1 × nonessential amino acids (NEAAs). All mammalian cell lines were grown in an atmosphere in the air with 5% CO_2_ at 37 ℃.

### Hamster brain tissues

Suckling Syrian golden hamsters (*Mesocricetus auratus*) at seven days old with clean health monitoring report (Janvier Labs, Le Genest-Saint-Isle, France) were used to prepare organotypic cultures as detailed elsewhere [65]. In brief, cerebella were isolated, and 350 μm-thick progressive slices were cut with a McIlwain tissue chopper (WPI-Europe). Each slice was then transferred and disrupted by vortexing in 350 µL of RA1 lysis buffer (Macherey–Nagel) containing 1% β-mercaptoethanol. This study was performed according to French ethical committee (CECCAPP) regulations (accreditation CECCAPP_ENS_2014_034).

### Viruses and plasmids

UUKV strain 23 was obtained from plasmids and amplified in BHK-21 cells [66]. The prototype strains of SFV, TOSV ISS, and GERV have been described previously [8, 67–69]. All viruses were produced in BHK-21 cells and purified and titrated according to standard procedures [8, 11, 32, 70]. Fluorescent labeling was performed with one molecule of viral glycoproteins per three molecules of ATTO488 dye (Atto-Tec) in 20 mM 4-(2-hydroxyethyl)-1-piperazineethanesulfonic acid (HEPES) following a protocol established by our group [32]. The MOI is presented based on the titers in BHK-21 cells. EBOVLPs were produced by transfecting HEK293T cells with plasmids encoding the EBOV structural proteins GP, VP40, and VP40-GFP at a 10:10:1 ratio. EBOVLPs were purified and concentrated as previously reported [71]. Briefly, supernatants from transfected cells were first cleared through successive centrifugation cycles at 4 °C. EBOVLPs were then pelleted in a 30%-sucrose cushion and resuspended in HNE buffer (10 mM HEPES, 100 mM NaCl, and 1 mM EDTA, pH 7.4). Residual sucrose was ultimately removed through a final ultracentrifugation step. The EBOVLP input was normalized based on the concentration of EBOV structural proteins as determined with a bicinchoninic acid (BCA) protein assay (Pierce™ BCA protein assay kit, Thermo Fisher Scientific). The simian virus 40 (SV40) promoter-driven expression vector encoding Gaussia luciferase, pFRTpsiCHECK_Gaussia_luciferase, was a gift from Markus Landthaler (Addgene plasmid # 154459, http://n2t.net/addgene:154459; RRID: Addgene_154459).

### Antibodies, small interfering RNAs (siRNAs), and other reagents

The mouse monoclonal antibody (mAb) 8B11A3 and the rabbit polyclonal antibodies (pAb) K1224 and K5 were directed against the UUKV nucleoprotein N and the glycoproteins Gn and Gc, respectively [72, 73]. All these antibodies were kind gifts from the Ludwig Institute for Cancer Research, Stockholm, Sweden. The rabbit pAb U2, which has been described previously, recognizes the UUKV proteins N, Gn, and Gc [12]. The mouse mAb E2-1 targets the SFV glycoprotein E2 and was a generous gift from M.C. Kielian (Albert Einstein College of Medicine) [74]. Mouse immune ascitic fluid recognizes all TOSV structural proteins and was a kind gift from R.B. Tesh, University of Texas [11]. The rabbit pAbs anti-actin C11, anti-calnexin CNX, anti-UDP-UGCG, and anti-GlcCer were purchased from Sigma Aldrich, LSBio, and Antibody Research, respectively. The mouse mAbs against TGN46 (2F7.1) and UGCG (1E5) were purchased from GeneTex and Santa Cruz Biotechnology, respectively. The anti-DC-SIGN phycoerythrin-conjugated fragment antigen binding (Fab) 1621P was purchased from R&D Systems. Nonoverlapping siRNAs against UGCG, β4Galt5, and β4Galt6 and siRNA negative control were designed in-house and obtained from Sigma-Aldrich. β4Galt5 and β4Galt6 each have two isoforms, and the siRNAs against the genes coding for these proteins target sequences common to both isoforms so that the two isoforms of each gene can be silenced with a single siRNA. The complete list of siRNAs is shown in Table S6. Soluble C6-GlcCer and C6-Cer were purchased from Cayman Chemical and dissolved in methanol. PPMP, PDMP, NB-DNJ, and NB-DGJ (Cayman Chemical), as well as brefeldin A (Sigma Aldrich), were also all dissolved in methanol. Ammonium chloride (NH_4_Cl, Sigma Aldrich) was prepared in water. PPMP, PDMP, NB-DNJ, NB-DGJ, and UUKV were assessed for cytotoxicity at the indicated amounts with a CytoTox96 nonradioactive cytotoxicity colorimetric assay kit (Promega) according to the manufacturer’s instructions. Endo H and PNGase F were purchased from New England Biolabs and used according to the manufacturer’s recommendations.

### mRNA and viral RNA quantification assays

RNA was harvested from infected cells and organotypic cultures using a NucleoSpin RNA extraction kit (Macherey–Nagel) as per manufactures instructions and cDNA was synthesized using PrimeScript™ RT Reagent (Takara) from 500 ng of total RNA according to supplier instructions. RT-qPCR was performed on 20 ng cDNA using TB green premix ExTaq (Takara) on AriaMx thermocycler (Agilent) following manufacturer recommendations. GUSB and Rpl22 were amplified and used as normalizing genes. Viral RNA was extracted from different volumes of virus stocks using RNAeasy RNA extraction kit (Qiagen) according to the provider’s protocol. cDNA was synthesized from same volumes of RNA using iSCRIPT reverse transcriptase (BioRad) as per manufacturer’s instructions. RT-qPCR was performed using 2 μL of sample per reaction using iTaq SYBR green (BioRad) following the manufacturer’s recommendations. For the analysis of virus stocks, primers that selectively target the viral RNA segments S, M, and L were used. The complete list of primers is shown in Table S7.

### siRNA-mediated silencing

siRNA transfections were performed with Lipofectamine RNAiMAX reagent according to the manufacturer’s protocol (Thermo Fisher Scientific) as reported previously [42]. Briefly, 25,000 cells were transfected with siRNAs at final concentrations of 20 nM and seeded in a 24-well plate 3 days before infection.

### Flow cytometry-based infection assay

Flow cytometry-based assays were performed according to standard procedures [12]. Briefly, cells were infected with viruses at the indicated MOIs in the absence of serum for 1 h at 37 ℃. The viral input was replaced with a serum-free medium, and the cells were incubated for up to 24 additional hours before fixation and permeabilization with 0.1% saponin. Infected cells were immunostained for detection of newly synthesized viral proteins with antibody 8B11A3 (anti-UUKV N), antibody 2E-1 (anti-SFV E2), and mouse ascitic fluid (TOSV) and quantified with FACS cytometers, namely Celesta, Canto, or Verse (all from Becton Dickinson) and FlowJo software (TreeStar). For the UGCG inactivation assay, cells were pretreated with drugs at the indicated concentrations for up to 24 h at 37 ℃ and then exposed to viruses in the continuous presence of inhibitors.

### Lipidomic analysis with MS

To analyze the lipid content of infected cells and viral particles, per experiment twelve T175 flasks of BHK-21 cells were exposed to viruses in the absence of serum for the indicated incubation periods before fixation with 100% methanol. Viral particles were first purified by centrifugation with a 25%-sucrose cushion and then a 15–60% sucrose gradient. Sucrose was removed from the viral stocks by additional ultracentrifugation and washing steps in HNE buffer before fixation with methanol. For inactivation of UGCG with PPMP, cells were pretreated with 2.5 µM drug for 16 h before exposure to UUKV for a further 24 h. Samples were subsequently subjected to lipid extraction in the presence of internal lipid standards and MS as previously described [75]. Lipid extraction was performed using an acidic liquid—liquid extraction method [76], except for plasmalogens, which were extracted under neutral conditions. To ensure that similar amounts of lipids were extracted, a test extraction was performed to determine the concentration of PC as a bulk membrane lipid. Quantification was achieved by adding 1–3 internal lipid standards with a structure similar to that of the endogenous lipid species representing each lipid class. Sample volumes were adjusted to ensure that the lipid standard-to-lipid species ratios were in a linear range for quantification with the standard-to-species ratios within a range of > 0.1 to < 10. Following this approach, the relative quantification of lipid species was performed. Typically, 1500−3000 pmol of total lipid was used for lipid extraction. Lipid standards were added prior to extraction; the master mix consisted of 50 pmol PC (13:0/13:0, 14:0/14:0, 20:0/20:0, and 21:0/21:0, Avanti Polar Lipids), 50 pmol SM (d18:1 with semi-synthesized N-acylated 13:0, 17:0, and 25:0 [77]), 100 pmol deuterated Chol (D7-Chol, Cambridge Isotope Laboratory), 30 pmol PI (17:0/20:4, Avanti Polar Lipids), 25 pmol PE and 25 pmol PS (both with semi-synthesized 14:1/14:1, 20:1/20:1, and 22:1/22:1 [77]), 25 pmol DAG (17:0/17:0, Larodan), 25 pmol CE (9:0, 19:0, and 24:1, Sigma), and 24 pmol TAG (LM-6000/D5-17:0,17:1, and 17:1, Avanti Polar Lipids), 5 pmol Cer (d18:1 with semi-synthesized N-acylated 14:0, 17:0, and 25:0 [77] or Cer (d18:1/18:0-D3, Matreya) and 5 pmol HexCer (d18:1 with semi-synthesized N-acylated 14:0, 19:0, and 27:0, or GlcCer (d18:1/17:0, Avanti Polar Lipids)), 5 pmol Hex2Cer (d18:1 with an N-acylated C17 fatty acid chain), 10 pmol PA (17:0/20:4, Avanti Polar Lipids), 10 pmol PG (with semi-synthesized 14:1/14:1, 20:1/20:1, and 22:1/22:1 [77]) and 5 pmol LPC (17:1, Avanti Polar Lipids). The PE P standard mix consisted of 16.5 pmol PE P-Mix 1 (16:0p/15:0, 16:0p/19:0, and 16:0p/25:0), 23.25 pmol PE P-Mix 2 (18:0p/15:0, 18:0p/19:0, and 18:0p/25:0), and 32.25 pmol PE P-Mix 3 (18:1p/15:0, 18:1p/19:0, and 18:1p/25:0). PE P was semi-synthesized as previously described in [78]. The final CHCl_3_ phase was evaporated under a gentle stream of nitrogen at 37 ℃. Samples were either directly subjected to MS analysis or were stored at – 20 ℃ before analysis, which was typically completed within 1–2 days of extraction. Lipid extracts were resuspended in 10 mM ammonium acetate in 60 µL of methanol. Two-microliter aliquots of resuspended lipids were diluted 1:10 in 10 mM ammonium acetate in methanol in 96-well plates (Eppendorf twin tec 96) before measurement. For Chol measurements, the remaining lipid extract was evaporated again and subjected to acetylation as previously described in [79]. Samples were analyzed on a QTRAP 6500 + mass spectrometer (Sciex) with chip-based (HD-D ESI Chip, Advion Biosciences) electrospray infusion and ionization Triversa Nanomate system (Advion Biosciences). In viral samples, TAG and DAG species were measured through a shotgun approach using a QExactive Plus instrument, and LipidXplorer software was used for data analysis as previously described in [17]. The data were evaluated by LipidView (Sciex and an in-house-developed software (ShinyLipids. Sphingolipid species annotation is presented as < number of total C atoms in the sphingosine backbone and N-acylated fatty acid > : < number of hydroxyl groups > , < number of double bonds > , and for all other lipids, the < number of total C atoms in acyl or alkenyl/alkanyl > : < number of double bonds > is reported.

### Protein analysis

Cells were lysed with 0.1% Triton X-100 or RIPA buffer (50 mM Tris − HCl, 1% Triton X-100, 0.05% SDS, 150 mM NaCl, 1 mM DTT, 1 mM EDTA, 1 mM protease inhibitor). Lysates were diluted in lithium dodecyl sulfate (LDS) sample buffer (Thermo Fisher Scientific), boiled for 5 min at 95 ℃, and analyzed by SDS-PAGE (Nu-PAGE Novex 4–12% or 10% Bis − Tris gels; Thermo Fisher Scientific). For Coomassie blue staining, gels were fixed in 50% (v/v) methanol and 10% (v/v) acetic acid and stained with the imperial protein stain (Thermo Fisher Scientific). For western blots, proteins were subsequently transferred to polyvinylidene difluoride (PVDF) membranes (iBlot Transfer Stacks; Thermo Fisher Scientific). The membranes were first blocked with intercept blocking buffer (LI-COR) or 5% milk and then incubated with primary antibodies against UGCG (LS Bio or Santa Cruz Biotechnology), UUKV structural proteins (U2), calnexin, TGN46, or actin, all diluted in Tris-buffered saline containing 0.1% Tween (TBS-T) and intercept blocking buffer or 5% milk. After extensive washing, the membranes were incubated with the corresponding secondary antibodies conjugated to IRDye 680RD or 800CW (LI-COR) or to horseradish peroxidase (HRP, Sigma Aldrich). Bound antibodies were exposed to enhanced chemiluminescent (ECL, Bio-Rad) and analyzed with an LI-COR Odyssey CLx scanner or a ChemiDoc XRS + scanner (Bio-Rad). Image processing was performed using ImageJ v1.52p (NIH) software and Image Studio™ software (LI-COR).

### Dot blot analysis

Cells were lysed in 0.1% Triton X-100, and lysates were added to nitrocellulose membranes using a Minifold^®^-1 Dot-Blot System (Whatman). The membranes were then immunostained with an antibody against GlcCer (111 586, Antibody Research) following the procedure described in the Protein analysis section.

### Luciferase-based assay

To examine whether the secretory pathway is intact, BHK-21 cells (10^5^) were transfected with 1 µg with the plasmid pFRTpsiCHECK_Gaussia_Luciferase using Lipofectamine 2000 (Thermo Fisher Scientific) according to the manufacturer’s instructions. One day post-transfection, cells were infected with UUKV at an MOI of 0.1 or treated with 2.5 µM PPMP or 1 µM Brefeldin A for a further 24 h. Luciferase activities were determined using the Dual-Luciferase Reporter Assay (Promega) following the supplier recommendations, and the luminescence signal was measured with a Varioskan Lux multimode reader (Thermo Fisher Scientific).

### Virus-binding and internalization assay

Viruses were allowed to bind to cells in binding buffer (DMEM or GMEM, pH 7.4, containing 0.2% BSA and 20 mM HEPES) on ice for up to 2 h, and binding was quantified by western blot, RT-qPCR, or flow cytometry analysis after extensive washing. Briefly, for the binding analysis of viruses produced in the absence or presence of GlcCer, the amount of input virus was normalized to the amount of viral nucleoprotein N, which correlated with the number of particles. The bound viruses were detected either by western blotting using the rabbit pAb U2 or by RT-qPCR targeting the S segment of the viral genome. To analyze binding competition by flow cytometry, soluble C6-GlcCer and C6-Cer at concentrations as high as 43.8 µM were prebound to cells on ice for 1.5 h before fluorescent particles were added at the indicated MOIs or GFP-containing EBOVLPs were added (500 ng per 10^5^ cells). In this series of assays, to inactivate UGCG, cells were pretreated with PPMP at 2.5 µM for 24 h at 37 °C before the binding of fluorescently-labeled virions. For internalization assays, cells were washed after virus binding and rapidly warmed to 37 °C for 30 min to trigger endocytosis. To distinguish between internalized and external particles, samples were treated with trypan blue (0.01%; Sigma) for 15 s at room temperature before flow cytometry analysis [8]. Binding and internalization were analyzed with either a FACS Celesta, Canto, or Verse flow cytometer (Becton Dickinson) and FlowJo software (TreeStar).

### DC-SIGN identification at the cell surface

The location of DC-SIGN was assessed at the surface of BHK-21 cells (not permeabilized) by flow cytometry using an anti-DC-SIGN phycoerythrin-conjugated antibody (FAB1621P R&D Systems) according to a standard procedure [64].

### Plunge freezing and cryo-electron tomography

To analyze UUKV particles produced in the presence or absence of PPMP, BHK-21 cells were pretreated either with 2.5 µM PPMP dissolved in methanol or with methanol for 16 h before exposure to UUKV at a low MOI (~ 0.1). The supernatant was harvested 24 h post-infection, and the virus particles were purified first through a 25%-sucrose cushion and then through a 15–60% sucrose gradient. The remaining sucrose was removed by additional ultracentrifugation and washing steps with HNE buffer. UUKV virions were then inactivated with 4% paraformaldehyde for biosafety reasons before plunge freezing with a Leica GP2 plunger at 80% humidity and a 25 ℃ air temperature. Viral suspensions were mixed with protein-A gold beads (10 nm), pipetted onto a 200 mesh copper grid coated with R2/1 Quantifoil carbon film, blotted from the backside of the mesh for 3 s with filter paper (Whatman No. 1), and plunge into frozen liquid ethane cooled to – 183 ℃. A tilt series was with a Krios cryo-transmission electron microscope at 300 keV (Thermo Fisher Scientific) equipped with a K3 direct electron detector and Quanta Imaging Filter (Gatan) with an energy slit set to 20 eV. A dose-symmetric tilt series obtained at increments of 3° and a tilt range of 120° was acquired with SerialEM 4.0 [80] at a magnification of 42,000 × (pixel size of 2.156 Å), defocus of -4 µm and electron dose per record of 3 e^−^/Å^2^. Projection images were aligned using fiducial gold and tomograms were reconstructed by weighted back projection in Etomo in the IMOD software package [81] using SIRT-like filter 5 and a dose-weighting filter.

### Statistical analysis

Prism v9.1.1 (GraphPad Software) was used for plotting numerical values in graphs and statistical analyses. The data are presented as the mean of independent experiments ± standard error of the mean (SEM) unless stated otherwise in the figure legends. The number of repeats, the statistical methods, and p values are indicated in figure legends when appropriate.

## Data and materials availability

All data are available in the main text, the supplementary materials, or will be made available on reasonable request.

### Supplementary Information

Below is the link to the electronic supplementary material.

Supplementary file1 Supplemental Table S1. Lipidomic analysis of BHK-21 cells infected with Uukuniemi virus (UUKV). This table is related to Figures 1A, 1D, and 1E (XLSX 47 KB)

Supplementary file2 Supplemental Table S2. Lipidomic analysis of purified Uukuniemi virus (UUKV) and Semliki forest virus (SFV) particles. This table is related to Figures 1C, 9B, S2A, and S5. (XLSX 53 KB)

Supplementary file3 Supplemental Table S3. Lipidomic analysis of Uukuniemi virus (UUKV)-infected BHK-21 cells in the presence of DL-threo-phenyl-2-palmitoylamino-3-morpholino-1-propanol (PPMP). This table is related to Figures 3A and 3B. (XLSX 35 KB)

Supplementary file4 Supplemental Table S4. Lipidomic analysis of purified Uukuniemi virus (UUKV) particles produced in the presence of PPMP. This table is related to Figure 3E (XLSX 30 KB)

Supplementary file5 Supplemental Table S5. Lipidomic analysis of BHK-21 cells infected with Semliki forest virus (SFV). This table is related to Figure 9A. (XLSX 30 KB)

Supplementary file6 Supplemental Table S6. Names and sequences of the siRNAs used in this study. (TIF 594 KB)

Supplementary file7 Supplemental Table S7. Names and sequences of the primers used for reverse transcription-quantitative PCR (RT-qPCR) in this study. a Forw., forward; Rev., reverse. (TIF 821 KB)

Supplementary file8 Supplemental Figure S1. Uukuniemi virus (UUKV) cytotoxicity in cells. BHK-21 cells were infected with UUKV at MOI ~0.1 for up to 48 h and assayed for cytotoxicity with a CytoTox96 non-radioactive cytotoxicity colorimetric assay kit (n = 2). (TIF 512 KB)

Supplementary file9 Supplemental Figure S2. Mass spectrometry (MS) analyses of Uukuniemi virus (UUKV) particles. (A) Supernatant from Uukuniemi virus (UUKV)-infected BHK-21 cells was harvested 48 h post-infection, and UUKV particles were purified before quantitative MS-based lipid analysis (n = 3). (B) Lysate, supernatant from infected BHK-21 cells used for UUKV production, and UUKV particles purified through a sucrose cushion were analyzed by reducing, SDS-PAGE and western blotting using antibodies against calnexin (CANX), TGN46, and UUKV proteins N, Gn, and Gc. (C) UUKV structural proteins from sucrose gradient-purified virus stocks were separated by nonreducing SDS-PAGE and stained with Coomassie blue. (TIF 1257 KB)

Supplementary file10 Supplemental Figure S3. DL-threo-phenyl-2-palmitoylamino-3-morpholino-1-propanol (PPMP) cytotoxicity in cells. (A to C) The cytotoxicity of four GlcCer inhibitors was determined using a CytoTox96 non-radioactive cytotoxicity colorimetric assay kit. Values were normalized to those of lysed, untreated cells, which corresponded to the maximum possible release of lactate dehydrogenase into the extracellular medium (n = 2). The inhibitors tested included (A) DL-threo-phenyl-2-palmitoylamino-3-morpholino-1-propanol (PPMP), (B) N-(2-hydroxy-1-(4-morpholinylmethyl)-2-phenylethyl)-decanamide (PDMP), and (C) N-butyl-deoxygalactonojirimycin (NB-DGJ) and N-butyl-deoxynojirimycin (NB-DNJ). The inhibitors were applied to A549 and BHK-21 cells for 24 h at varying concentrations as indicated. A concentration of 50 μM PPMP was used as a positive control (ctrl). (TIF 611 KB)

Supplementary file11 Supplemental Figure S4. DL-threo-phenyl-2-palmitoylamino-3-morpholino-1-propanol (PPMP) treatment reduces UUKV infection in A549 cells. A549 lung epithelial cells were pretreated with PPMP for 16 h and then exposed to UUKV (multiplicity of infection ~2) in the continuous presence of the inhibitor. Infected cells were harvested 8 h later and immunostained for UUKV nucleoprotein N. Infection was analyzed by flow cytometry, and the data were normalized to those of cells infected in the absence of the inhibitor; i.e., it was reported as the percentage of the control. One-way ANOVA with Dunnett’s multiple comparison test was applied (n = 2). *, p < 0.05; ns, not significant. (TIF 502 KB)

Supplementary file12 Supplemental Figure S5. Mass spectrometry (MS) analyses of Semliki forest virus (SFV) particles. Supernatant from SFV-infected BHK-21 cells was harvested 24 h post-infection, and SFV particles were purified before quantitative MS-based lipid analysis (n = 3). (TIF 521 KB)
