# Supplementary figures and images for: Glucosylceramide in bunyavirus particles is essential for virus binding to host cells
Source: Cell Mol Life Sci. 2024 Feb 1;81(1):71. doi: 10.1007/s00018-023-05103-0 (PMC10834583; doi:10.1007/s00018-023-05103-0)

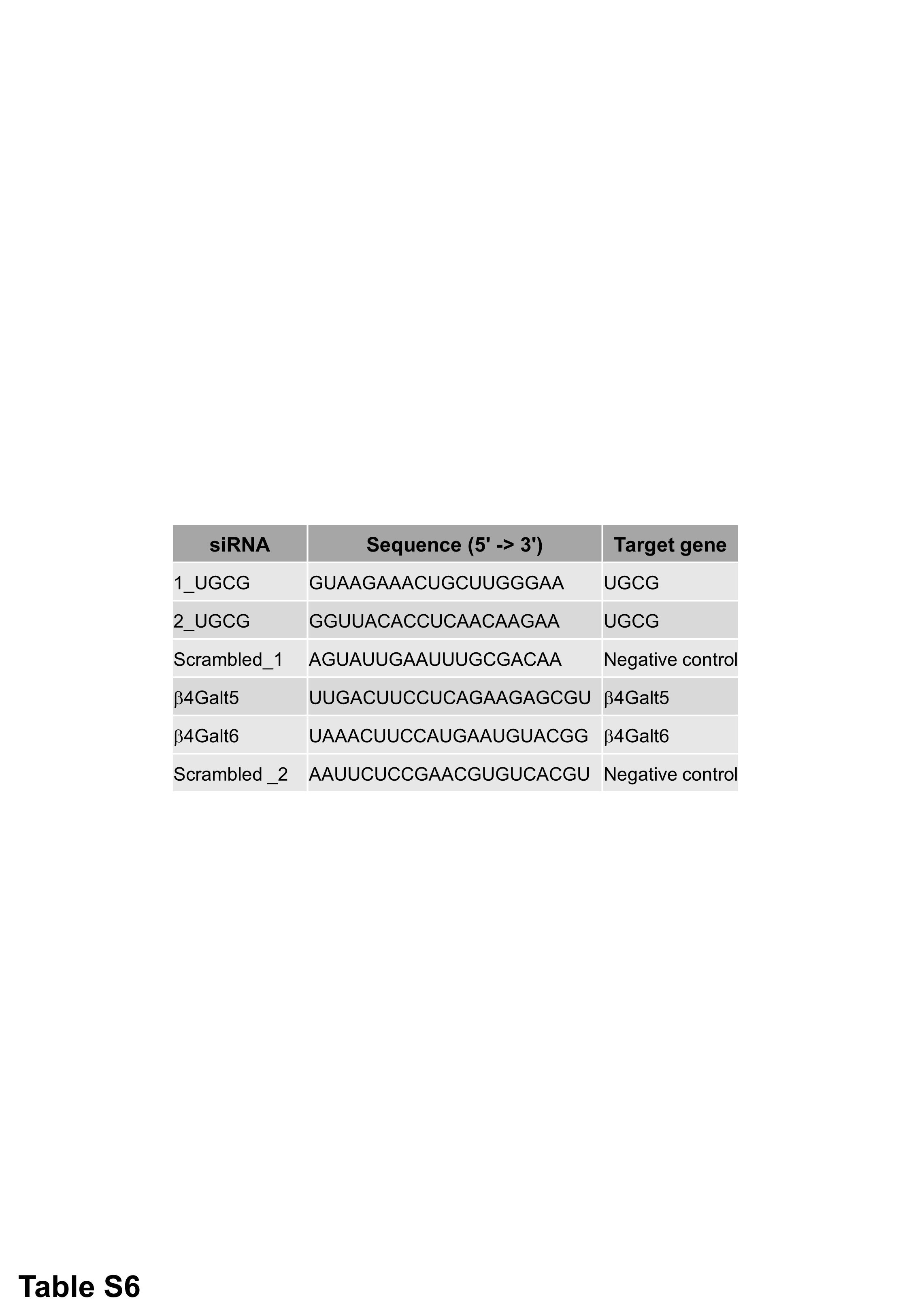

Supplement: Supplementary file 6 — Supplementary file6 Supplemental Table S6. Names and sequences of the siRNAs used in this study. (TIF 594 KB) [file 18_2023_5103_MOESM6_ESM.tif]

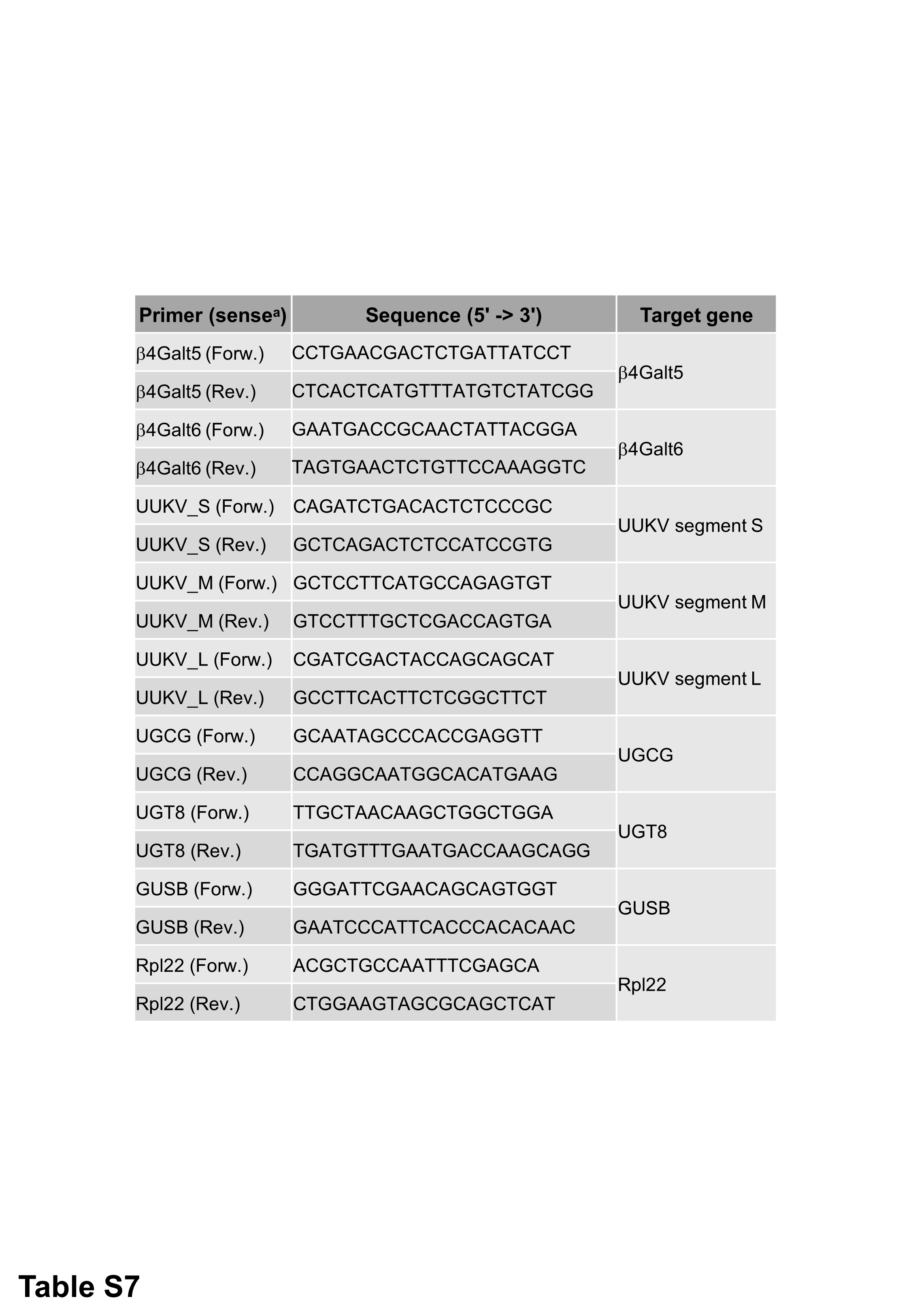

Supplement: Supplementary file 7 — Supplementary file7 Supplemental Table S7. Names and sequences of the primers used for reverse transcription-quantitative PCR (RT-qPCR) in this study. a Forw., forward; Rev., reverse. (TIF 821 KB) [file 18_2023_5103_MOESM7_ESM.tif]

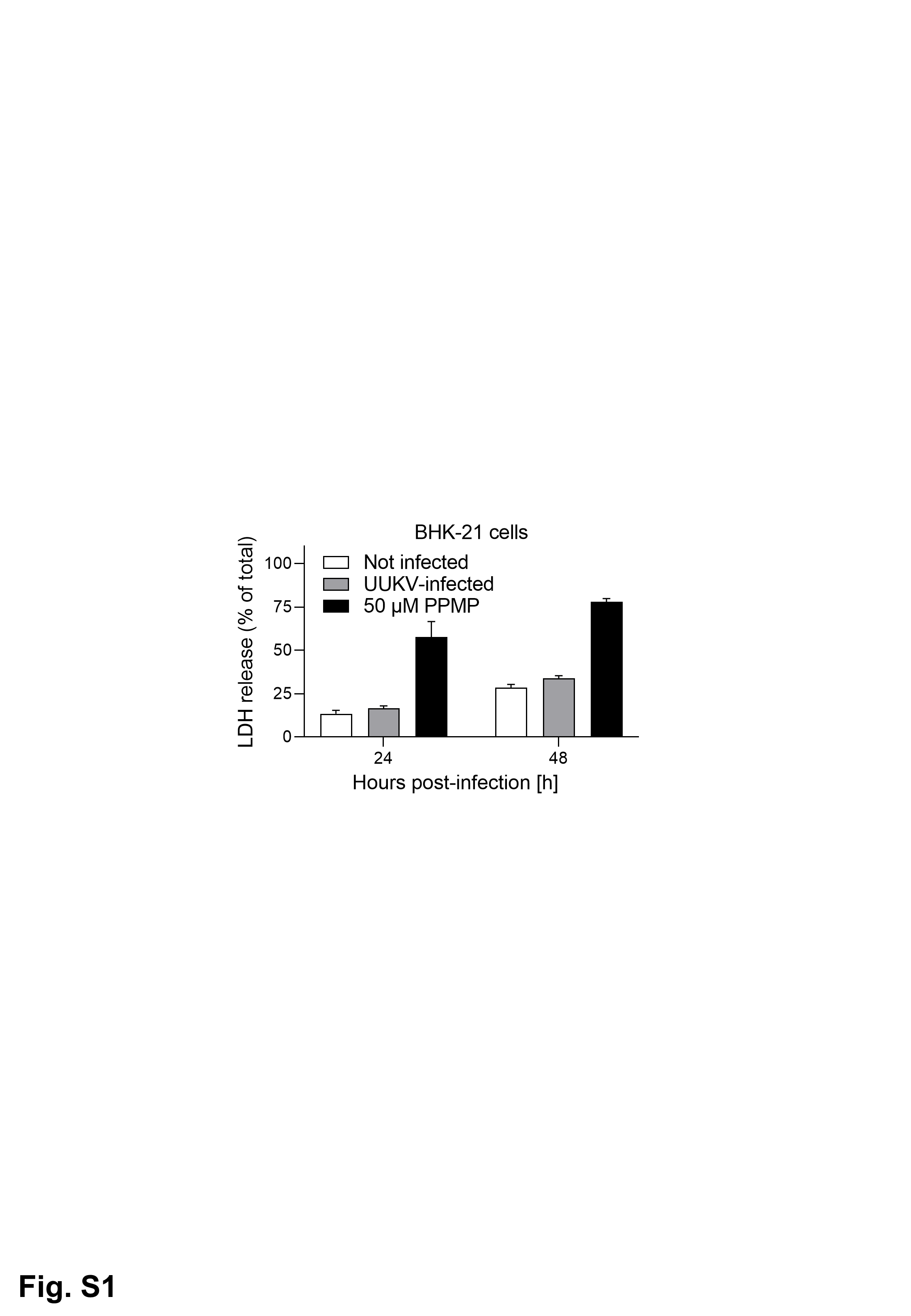

Supplement: Supplementary file 8 — Supplementary file8 Supplemental Figure S1. Uukuniemi virus (UUKV) cytotoxicity in cells. BHK-21 cells were infected with UUKV at MOI ~0.1 for up to 48 h and assayed for cytotoxicity with a CytoTox96 non-radioactive cytotoxicity colorimetric assay kit (n = 2). (TIF 512 KB) [file 18_2023_5103_MOESM8_ESM.tif]

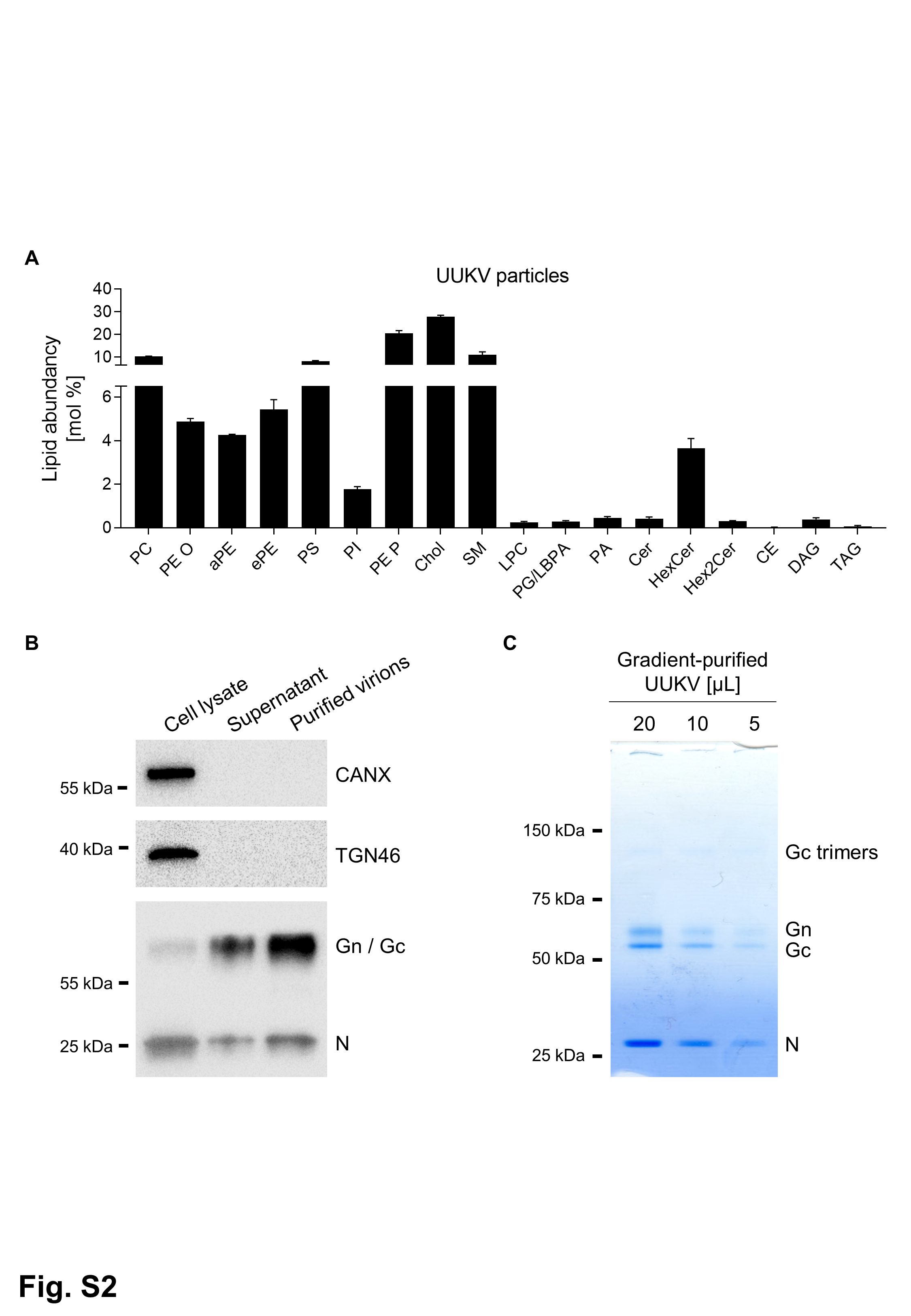

Supplement: Supplementary file 9 — Supplementary file9 Supplemental Figure S2. Mass spectrometry (MS) analyses of Uukuniemi virus (UUKV) particles. (A) Supernatant from Uukuniemi virus (UUKV)-infected BHK-21 cells was harvested 48 h post-infection, and UUKV particles were purified before quantitative MS-based lipid analysis (n = 3). (B) Lysate, supernatant from infected BHK-21 cells used for UUKV production, and UUKV particles purified through a sucrose cushion were analyzed by reducing, SDS-PAGE and western blotting using antibodies against calnexin (CANX), TGN46, and UUKV proteins N, Gn, and Gc. (C) UUKV structural proteins from sucrose gradient-purified virus stocks were separated by nonreducing SDS-PAGE and stained with Coomassie blue. (TIF 1257 KB) [file 18_2023_5103_MOESM9_ESM.tif]

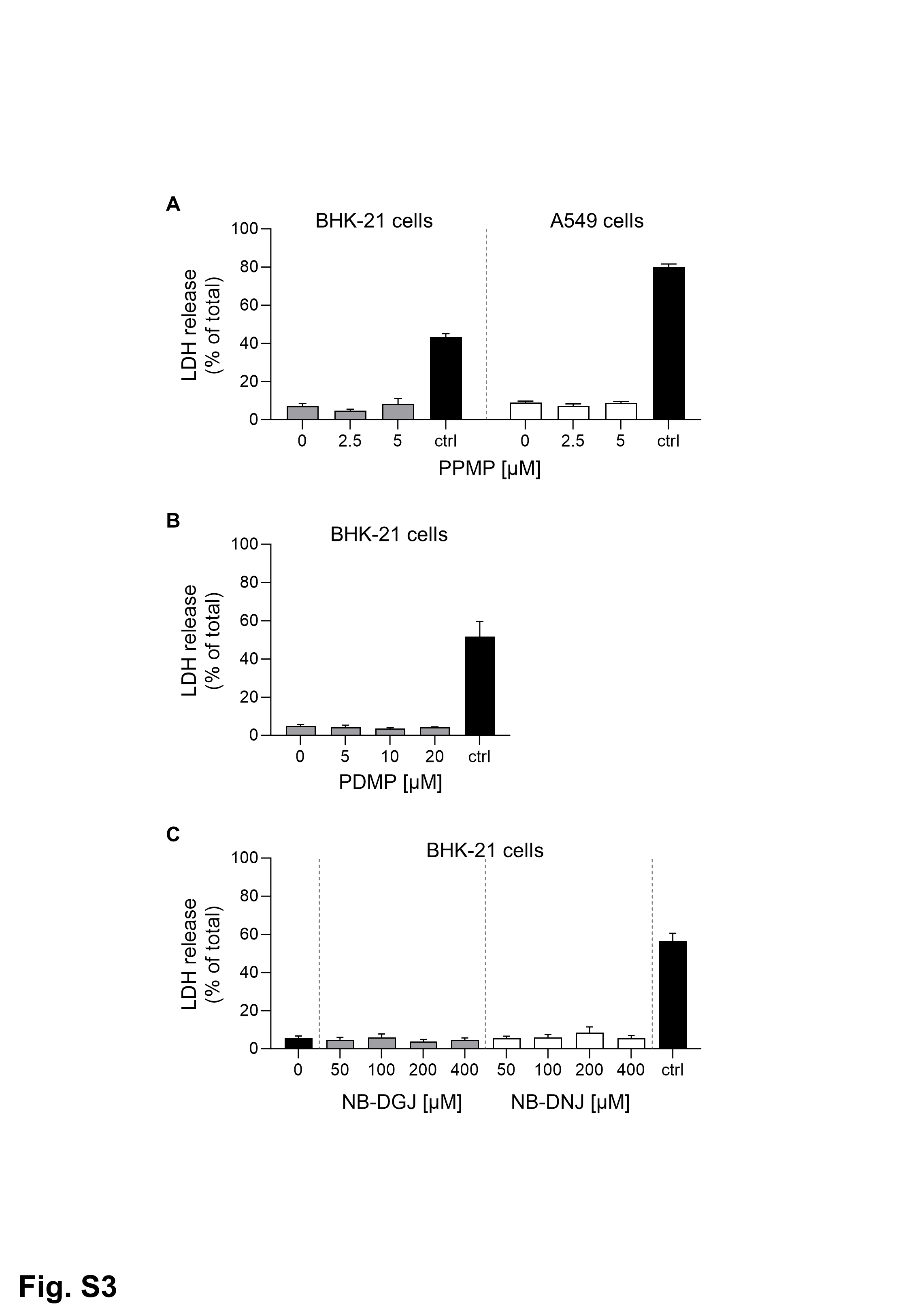

Supplement: Supplementary file 10 — Supplementary file10 Supplemental Figure S3. DL-threo-phenyl-2-palmitoylamino-3-morpholino-1-propanol (PPMP) cytotoxicity in cells. (A to C) The cytotoxicity of four GlcCer inhibitors was determined using a CytoTox96 non-radioactive cytotoxicity colorimetric assay kit. Values were normalized to those of lysed, untreated cells, which corresponded to the maximum possible release of lactate dehydrogenase into the extracellular medium (n = 2). The inhibitors tested included (A) DL-threo-phenyl-2-palmitoylamino-3-morpholino-1-propanol (PPMP), (B) N-(2-hydroxy-1-(4-morpholinylmethyl)-2-phenylethyl)-decanamide (PDMP), and (C) N-butyl-deoxygalactonojirimycin (NB-DGJ) and N-butyl-deoxynojirimycin (NB-DNJ). The inhibitors were applied to A549 and BHK-21 cells for 24 h at varying concentrations as indicated. A concentration of 50 μM PPMP was used as a positive control (ctrl). (TIF 611 KB) [file 18_2023_5103_MOESM10_ESM.tif]

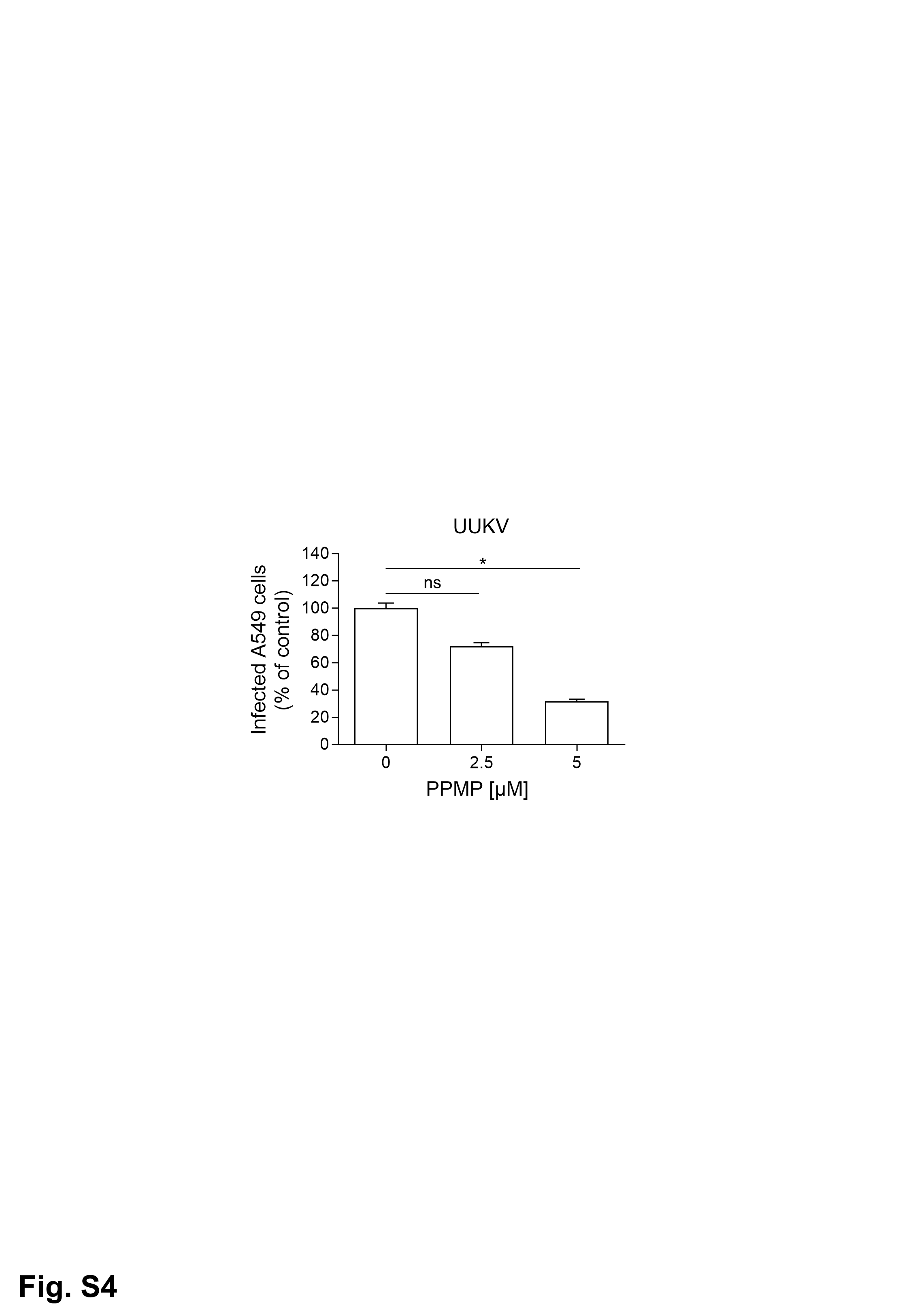

Supplement: Supplementary file 11 — Supplementary file11 Supplemental Figure S4. DL-threo-phenyl-2-palmitoylamino-3-morpholino-1-propanol (PPMP) treatment reduces UUKV infection in A549 cells. A549 lung epithelial cells were pretreated with PPMP for 16 h and then exposed to UUKV (multiplicity of infection ~2) in the continuous presence of the inhibitor. Infected cells were harvested 8 h later and immunostained for UUKV nucleoprotein N. Infection was analyzed by flow cytometry, and the data were normalized to those of cells infected in the absence of the inhibitor; i.e., it was reported as the percentage of the control. One-way ANOVA with Dunnett’s multiple comparison test was applied (n = 2). *, p < 0.05; ns, not significant. (TIF 502 KB) [file 18_2023_5103_MOESM11_ESM.tif]

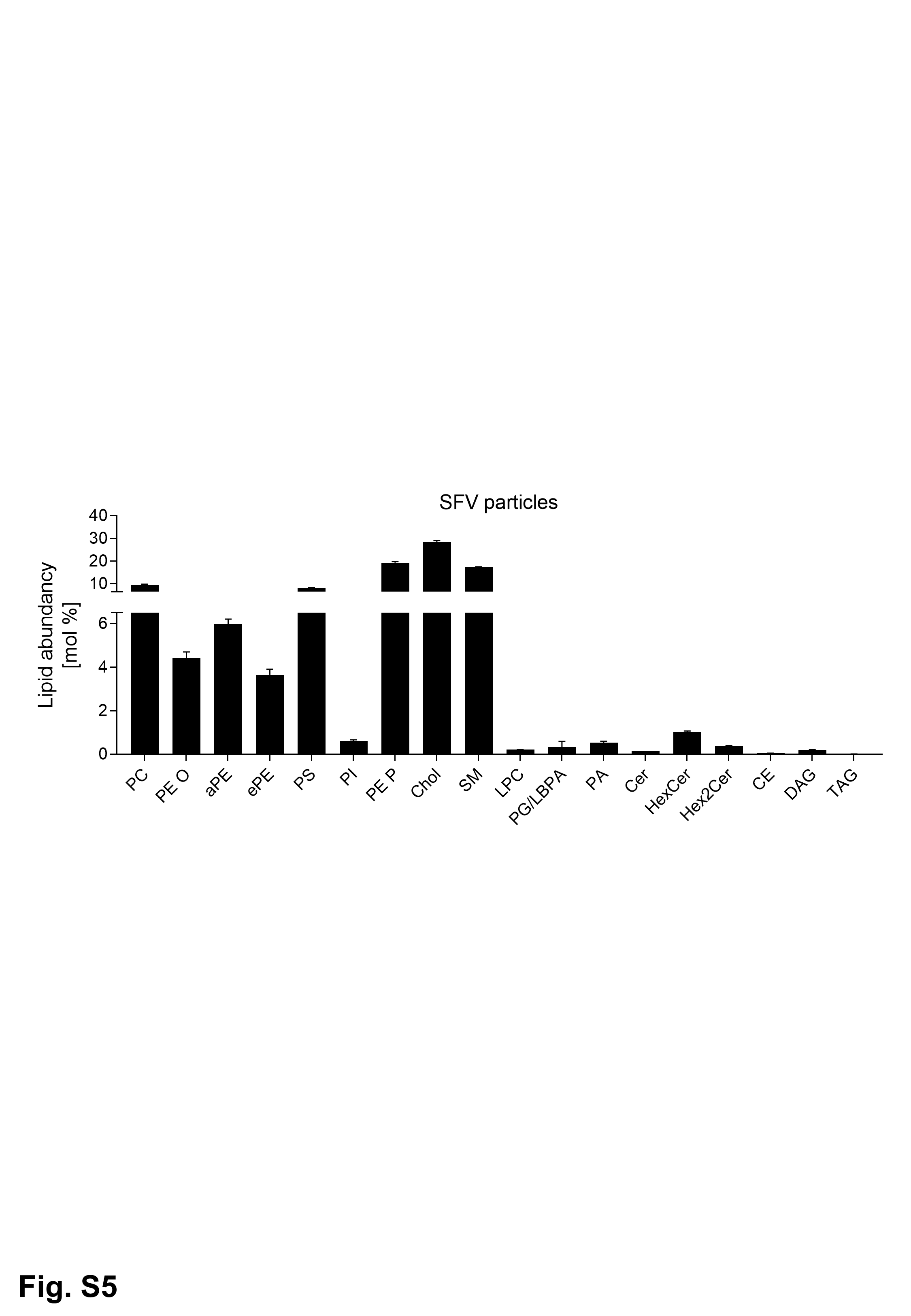

Supplement: Supplementary file 12 — Supplementary file12 Supplemental Figure S5. Mass spectrometry (MS) analyses of Semliki forest virus (SFV) particles. Supernatant from SFV-infected BHK-21 cells was harvested 24 h post-infection, and SFV particles were purified before quantitative MS-based lipid analysis (n = 3). (TIF 521 KB) [file 18_2023_5103_MOESM12_ESM.tif]
